# Detection of the Na_v_ channel *kdr*-like mutation and modeling of factors affecting survivorship of *Culex quinquefasciatus* mosquitoes from six areas of Harris County (Houston), Texas, after permethrin field-cage tests

**DOI:** 10.1371/journal.pntd.0008860

**Published:** 2020-11-19

**Authors:** Han-Jung Lee, Michael Longnecker, Travis L. Calkins, Andrew D. Renfro, Chris L. Fredregill, Mustapha Debboun, Patricia V. Pietrantonio

**Affiliations:** 1 Department of Entomology, Texas A&M University, College Station, Texas, United States of America; 2 Department of Statistics, Texas A&M University, College Station, Texas, United States of America; 3 Harris County Public Health, Mosquito and Vector Control Division (HCPH-MVCD), Texas, United States of America; Instituto de Pesquisas Veterinarias Desiderio Finamor, BRAZIL

## Abstract

*Culex quinquefasciatus* is one of the most important mosquito vectors of arboviruses. Currently, the fastest approach to control disease transmission is the application of synthetic adulticide insecticides. However, in highly populated urban centers the development of insecticide resistance in mosquito populations could impair insecticide efficacy and therefore, disease control. To assess the effect of resistance on vector control, females of *Cx*. *quinquefasciatus* collected from six mosquito control operational areas in Harris County, Texas, were treated in field cage tests at three different distances with the pyrethroid Permanone^®^ 31–66 applied at the operational rate. Females were analyzed by sequencing and/or diagnostic PCR using de novo designed primers for detecting the *kdr*-like mutation in the voltage-gated sodium channel (L982F; TTA to TTT) (house fly *kdr* canonical mutation L1014F). Females from the *Cx*. *quinquefasciatus* susceptible Sebring strain and those from the six operational areas placed at 30.4 m from the treatment source were killed in the tests, while 14% of field-collected mosquitoes survived at 60.8 m, and 35% at 91.2 m from the source. The diagnostic PCR had a with 97.5% accuracy to detect the *kdr*-like mutation. Pyrethroid resistant mosquitoes carrying the L982F mutation were broadly distributed in Harris County at high frequency. Among mosquitoes analyzed (n = 1,028), the *kdr*-*kdr* genotype was prevalent (81.2%), the *kdr*-s genotype was 18%, and s-s mosquitoes were less than 1% (n = 8). A logistic regression model estimated an equal probability of survival for the genotypes *kdr*-*kdr* and *kdr*-s in all areas analyzed. Altogether, our results point to a high-risk situation for the pyrethroid-based arboviral disease control in Harris County.

## Author summary

Female *Culex quinquefasciatus* mosquitoes blood-feed preferentially on birds but also on mammals including humans, to which they may transmit pathogenic arboviruses. To prevent transmission of West Nile and/or other viruses in Harris County, TX, the vector control agency applies mosquito adulticides when arboviruses are detected. Insecticide resistance can hinder mosquito control, increasing the risk of disease transmission. Pyrethroid resistant mosquitoes carry *kdr*-like mutation(s) in the sodium channel, the pyrethroid target site in the nervous system, that increases survival probability. We investigated frequency and spatial distribution of the *kdr*-like mutation in Harris County mosquitoes, and how they affect pyrethroid survivorship. We conducted field cage tests with a commercial pyrethroid formulation, and developed molecular tests to detect the *kdr*-like mutation in females from 6 operational areas. In field tests, almost no mosquito survived at 30.4 m, but 14% and 35% of mosquitoes survived at 60.8 m and 91.2 m. We found 99% of mosquitoes carried at least one *kdr*-like allele. At the high rate of pyrethroid, the survival probability of mosquitoes carrying one or two *kdr*-like alleles was not statistically different in the six areas, but survival increased significantly with distance. These results should be considered when analyzing risk of disease transmission.

## Introduction

In the southern United States (US), *Culex quinquefasciatus* Say (Diptera: Culicidae) is a vector for various arboviruses, including West Nile virus (WNV), Saint Louis Encephalitis virus (SLEV), and Eastern Equine Encephalitis virus (EEEV) [1,2]. In Texas (TX), *Cx*. *quinquefasciatus* is found in 150 counties, being the predominant mosquito [3] and its wide distribution contributes to the spread of arboviral diseases. From 2002 to 2016, 5,277 WNV human cases, and in 2017, 135 human WNV cases and 6 deaths were reported by the Texas Department of State Health Services (DSHS).

Harris County, TX, includes Houston, the fourth-largest city in the US, and has an estimated population of 4.6 million residents. It was also the leading destination for the 2.5 million new residents of TX in the last five years [4]. WNV was yearly detected in this county since it was first found in 2002. In 2014, the WNV outbreak caused 139 cases and 2 deaths [5]. Harris County is also under the threat of EEEV, detected in the adjacent Galveston County in 2017 as reported by DSHS. To protect the health of residents from arboviral diseases, it is crucial to control mosquito populations. Harris County Public Health, Mosquito and Vector Control Division (HCPH-MVCD), utilizes different mosquito control approaches including insecticide applications and the release of sterilized mosquitoes and of natural enemies against immatures, e.g. the entomopathogenic nematode *Romanomermis culicivorax*.

When WNV is detected in any of the 268 mosquito control operational areas, HCPH-MVCD applies insecticides weekly in that area, alternating malathion (Fyfanon^®^) and a permethrin-based adulticide (Permanone^®^ 31–66 or Evoluer^®^ 31–66) on a County-base rotation, e.g. operational areas where WNV is detected are treated with the first insecticide on odd weeks and with the second on even weeks. Because malathion is an organophosphate (OP) pesticide that inhibits acetylcholinesterase, and permethrin is a pyrethroid pesticide which targets the voltage-gated sodium channel (Na_v_) [6], it is expected that the alternate use of these adulticides with different modes of action will delay the development of resistance. Surveillance of the susceptibility status of the population and the identification of the mechanism(s) of resistance to the applied adulticides are still crucial to manage a successful vector control program. Previously, Pietrantonio et al. (2000) discovered that *Cx*. *quinquefasciatus* mosquitoes collected from 8 operational areas in Harris County, numbers 42, 51, 54, 55, 66, 106, 206, and 512, were highly resistant to malathion. The percent survival of females after exposure to malathion was 77–100%. Higher esterase activity, the major mechanism of resistance to OPs, was detected in the females collected from these 8 operational areas. Most of these females were still susceptible to Scourge^®^, a resmethrin (pyrethroid) based pesticide, with a percent survival of 0–20% [7]. Stark et al. (2017) and Dennett et al. (2017) performed field cage tests and revealed that 96% *Cx*. *quinquefasciatus* collected from 8 operational areas, 51, 55, 93, 109, 205, 225, 903 and 904, were killed by Evoluer^®^ 31–66 [8,9].

**Fig 1:**
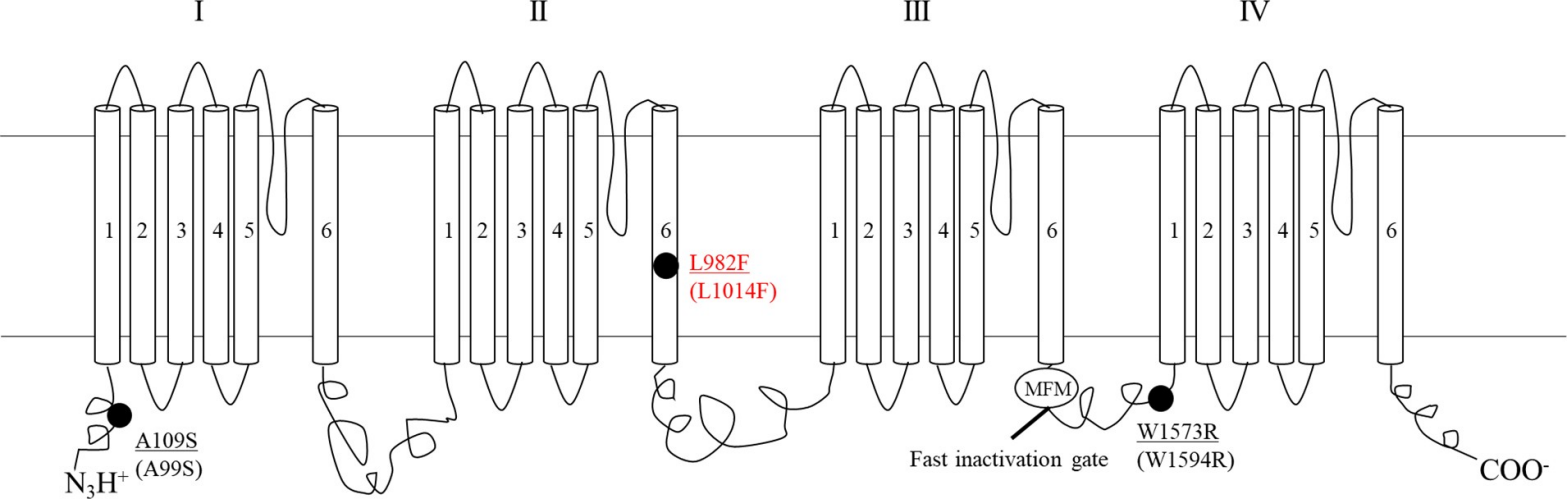
Pyrethroid-resistance associated SNPs in the voltage-gated sodium channel (VGSC, Na_v_) of *Culex quinquefasciatus*. The solid circles represent non-synonymous mutations. Other synonymous mutations associated with resistance have been identified but are not shown here. For each mutation site shown, the top is the mutation site in *Cx*. *quinquefasciatus*. The bottom number (in parenthesis) is the mutation site in *Musca domestica*. The *kdr*-like mutation analyzed here is in red [11].

The major mechanisms that cause pyrethroid resistance in *Culex* mosquitoes are target site insensitivity of the voltage-gated sodium channel (Na_v_) (Fig 1) and hyper-detoxification ability [10]. Pyrethroids bind to the Na_v_ preventing channel closure and inhibiting neuron repolarization [6]. The knock-down resistant (*kdr*) mutation is one of the most studied target site insensitivity mutations caused by single nucleotide polymorphisms (SNPs) that render mosquitoes resistant to pyrethroids (Fig 1) [11,12]. This mutation is located in domain II segment 6 (IIS6), and changes leucine to phenylalanine (“L to F”), serine (“L to S”) or histidine (“L to H”) [11,13–15]. These substitutions lower the binding affinity of pyrethroids to the Na_v_.

The molecular mechanisms that contribute to pyrethroid resistance of *Cx*. *quinquenfasciatus* in Harris County have not been fully investigated yet. Therefore, the susceptibility status of females to pyrethroid insecticide was evaluated through field cages placed at three different distances from the moving pyrethroid source, simulating a real control scenario. Subsequently, all field cage tested mosquitoes were analyzed with a diagnostic PCR we developed, and/or by sequencing of the sodium channel region encompassing the *kdr-*like mutation to confirm the PCR diagnostics. One primer set was designed for allele-specific PCR (AS-PCR) for detecting the *kdr*-like mutation. Analysis of the factors contributing to mortality in the field included a logistic regression model.

## Materials and methods

### Mosquitoes and field cage tests

**Fig 2:**
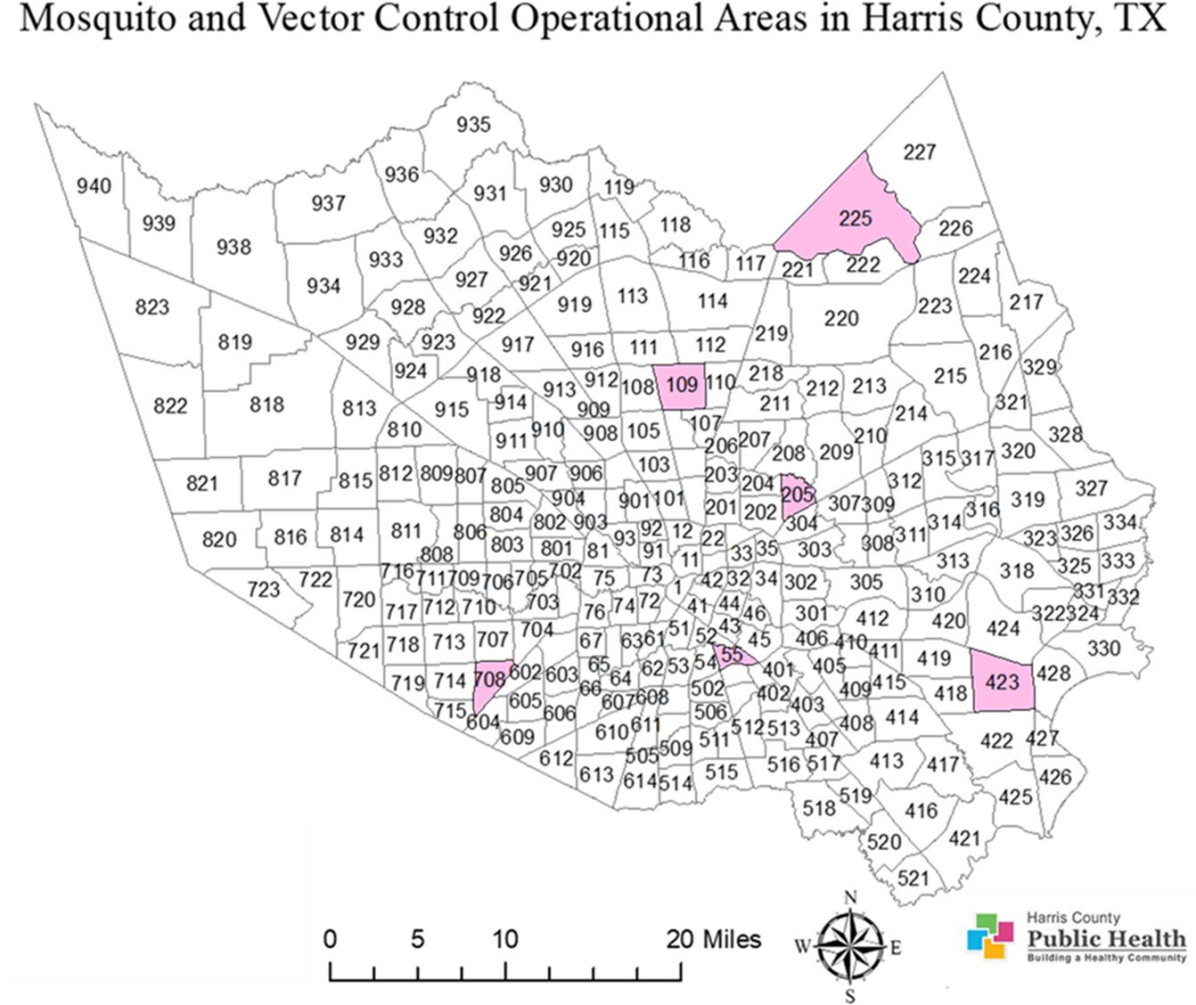
Map of 268 mosquito control operational areas in Harris County. The six operational areas labeled in pink are where egg rafts of *Cx*. *quinquefasciatus* were collected for female field cage pyrethroid tests. This map shows the Harris County boundary (map image layer by PHES_AGO; created Jun 7, 2017, updated May 7, 2020) and operational area boundaries (map image layer by PHES_AGO; created Nov 4, 2016, updated May 9, 2020). The layers for county (Map service: Harris county boundary masked) (https://www.arcgis.com/home/item.html?id=a8aa2ef4067348c79ccea62857a2f623) and for Harris County operational area boundaries (MVCD_Operational_Areas) (https://www.arcgis.com/home/item.html?id=66643535e01b42d3aae5d4647f5e1a6c) were created with ArcGIS (https://www.arcgis.com/home/webmap/viewer.html; ESRI, CA) by Harris County Public Health and are publicly available. There are no special restrictions or limitations on the terms of use of the layers applied to this map. The map was completed by assembling these two layers and by marking and coloring the research areas using the ArcMap 10.5 software (ESRI, CA).

Egg rafts of *Cx*. *quinquefasciatus* Say mosquitoes were collected in the field in 2018 and reared in the laboratory to obtain sufficient adults for insecticide resistance tests. A minimum of 300 egg rafts were collected by ovitraps filled with grass-fermented water from six insecticide-treated areas out of the 268 operational areas in Harris County. In each operational area, the egg rafts were obtained from six to fifteen different sites to ensure mosquito population diversity; egg rafts were collected from areas 55, 109, 205, 225, 423, and 708 (Fig 2). Among these areas, the first four were chosen by HCPH-MVCD for long-term insecticide resistance monitoring. Areas 423 and 708 were randomly selected among the operational areas that were positive for West Nile virus (WNV) in 2017. Eggs were hatched and mosquitoes were reared in the laboratory at 28–29.4°C. After adult emergence, two- to five-day-old females were separated from males using an aspirator. Females were placed in cages for tests to determine their survivorship in field insecticide applications; other details are as described [8]. The Sebring insecticide-susceptible laboratory strain of *Cx*. *quinquefasciatus* was maintained in the laboratory for females to be used as controls in the field tests.

**Fig 3:**
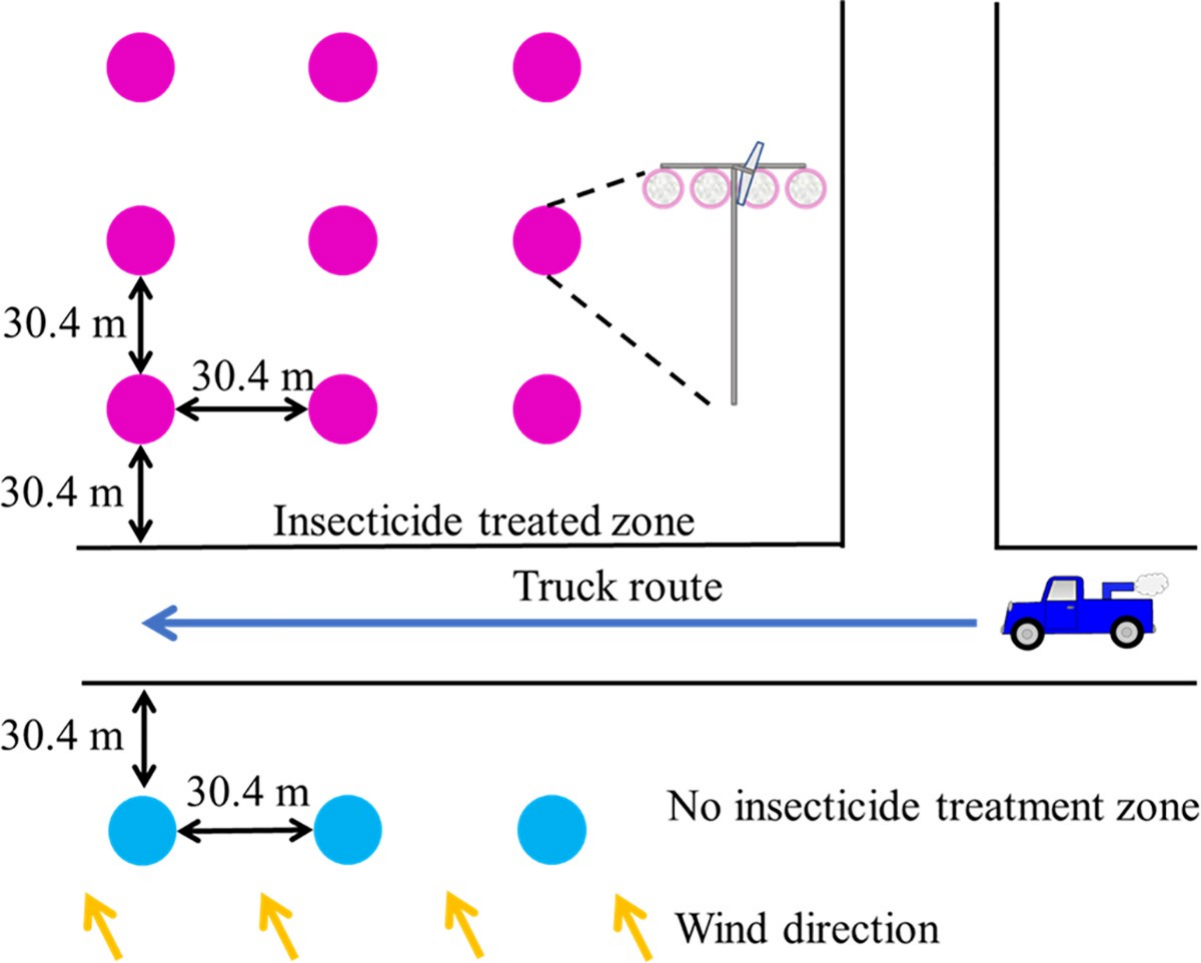
Schematic of the setup of the field cage test for insecticide effectiveness. Both magenta and blue dots represent a pole that holds three cages containing females reared from field-collected eggs from the three respective operational areas, and one cage containing Sebring strain susceptible mosquitoes. Each pole also has a flap to correct the orientation of cages for wind-direction (insert, right). Three poles are placed at 30.4 m, 60.8 m and 91.2 m (magenta dots), from the road in which the moving truck (~16 kph) applies the insecticide. Cages from poles represented by the blue dots serve as the negative control, because this zone should be a non-kill zone, upwind of the predominant wind direction.

Two tests were performed, on July 2018 and August 2018, to test females’ susceptibility to the pyrethroid Permanone^®^ 31–66 (a.i. permethrin, 31.28%, and synergist piperonyl butoxide, 66%; Bayer Environmental Science, Cary, NC). Posts with hanging cages, each containing 20–25 females, were placed at three different distances from the Permanone^®^ treatment source (Fig 3). Within the same row, each pseudoreplicate consisted of a post with a transverse bar with four hanging cages displayed horizontally at 1.5 m from the ground. One cage had Sebring strain females and each of three other cages had females obtained from field-collected eggs from the three selected operational areas, respectively (Fig 2). Three pseudoreplicates were utilized for each of three downwind distances (30.4 m, 60.8 m, and 91.2 m; magenta dots, Fig 3) from the route of a truck applying Permanone^®^ 31–66 (0.006 kg a.i./ha) [8], simulating a normal insecticide application (Fig 3). Additional mosquitoes used as negative controls were located in a row 30.4 m upwind of the truck route (blue dots, Fig 3), including three pseudoreplicates of the same cage sets as described in the downwind treatment. The cages placed downwind were expected to receive the insecticide treatment, while the upwind controls were not, due to the predominant wind direction and speed. Both of these variables are monitored before and during the assay. After the insecticide treatment, mosquitoes were anesthetized by delivering CO_2_, promptly transferred to holding cups, and carried back to the laboratory in coolers. The holding cups were hung on a wire rack in the laboratory and the evaluation of mortality was after 12 h. After mortality evaluation, the mosquitoes were placed in tubes, labeled, and transported under dry ice to the Insect Toxicology Laboratory at Texas A&M University (TAMU) where they were stored at -20°C until molecular diagnosis.

### Genomic DNA extraction of tested mosquitoes

Genomic DNA (gDNA) was extracted from field-tested mosquitoes, both from the Sebring susceptible strain (treated controls downwind) and those from the different operational areas, using the Quick-DNA^TM^ microprep kit (Zymo Research, Irvine, CA) following the manufacturer’s protocol with slight modifications. In brief, individual females were first surface-sterilized with 70% ethanol and transferred into 1.5 ml microcentrifuge tubes with 100 μl of genomic lysis buffer, and homogenized, either manually with a pestle, or mechanically in a TissueLyser II (Qiagen, Germantown, MD). The latter was done using 0.318 cm diameter stainless steel beads for 45 s at room temperature, followed by a 1 min centrifugation at 3,500 x *g* to eliminate bubbles, and another homogenization for 45 s. Genomic lysis buffer (400 μl) was added. Samples were incubated for 1 h at room temperature before centrifugation to ensure maximal yield, because storage at -20°C had caused mosquito dehydration. Samples were centrifuged at 10,000 x *g* for 5 min to remove wings, remaining tissues or exoskeleton. The supernatant was transferred to the Zymo-Spin^TM^ IC column and centrifuged at 10,000 x *g* for 1 min. Columns were transferred into a new collection tube and 200 μl of DNA Pre-Wash Buffer was added before centrifugation at 10,000 x *g* for 1 min. Before a second centrifugation at 10,000 x *g* for 1 min, 500 μl of gDNA Wash Buffer was added to the spin column. Spin columns were transferred to clean 1.5 ml tubes, 20 μl of nuclease free water was added to the column filter, and the column was incubated for 5 min at room temperature before the final centrifugation at 10,000 x *g* for 1 min. The eluted gDNA was quantified with a Nanoquant M200 Pro (Tecan, Morrisville, NC), and was stored at 4°C for 1–120 days before diagnostic PCR was performed.

### Development of an allele-specific diagnostic PCR for the L982F *kdr*-like mutation

**Fig 4:**
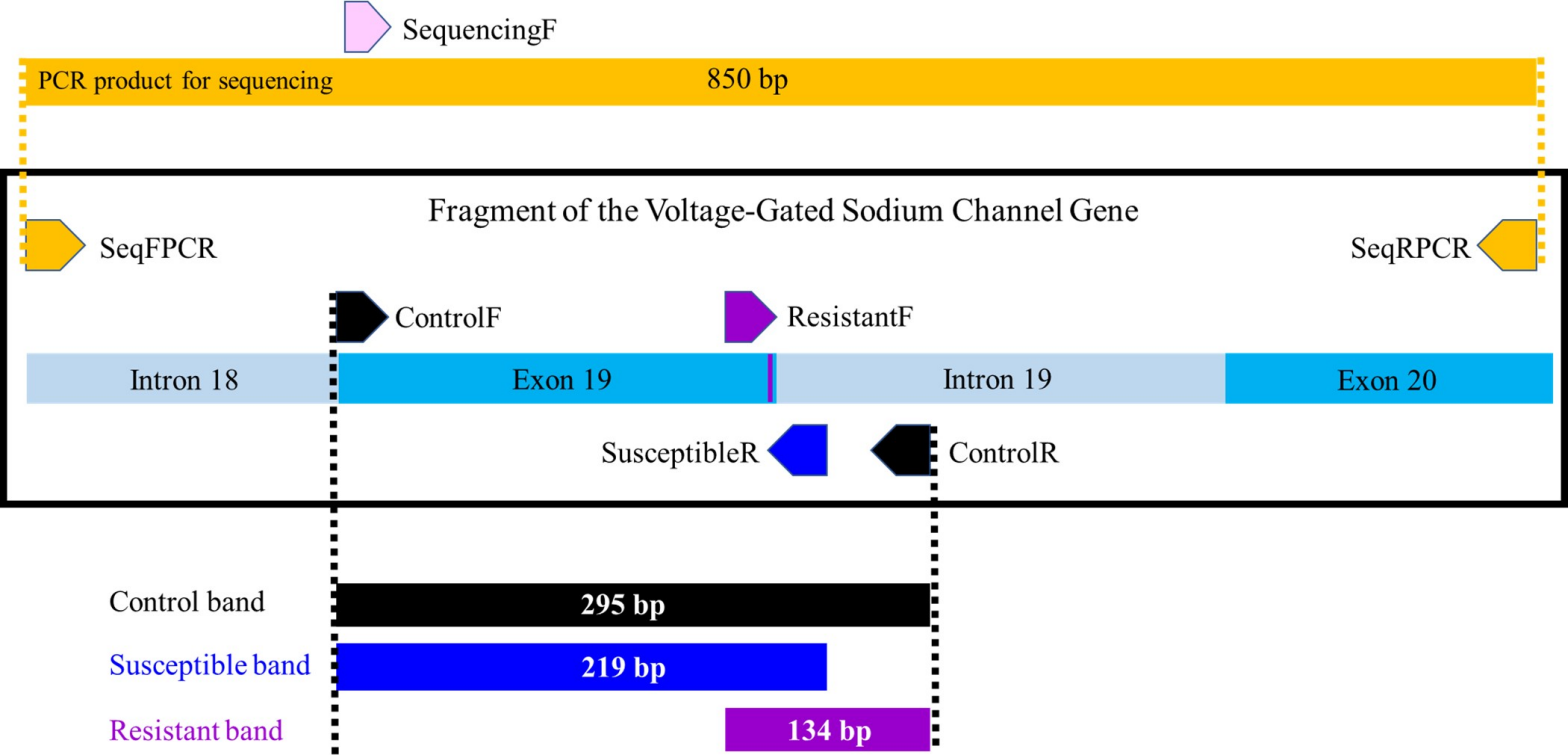
Schematic primer map and expected PCR products of the genomic *kdr* region. The schematic is shown in the genomic forward orientation for clarity. The exon and intron numbers in the light blue boxes are according to the sodium channel sequence of the S-Lab strain mosquito published by He et al. (2012) [17]. The *kdr*-like mutation (L982F) is marked as vertical purple line on Exon19. The expected product sizes of diagnostic PCR: 295 bp control band in black is generated by ControlF and ControlR; 219 bp susceptible band in dark blue is generated by ControlF and SusceptibleR; 134 bp resistant band in purple is generated by ResistantF and ControlR. The 850 bp PCR product generated by primers in orange (SeqFPCR and SeqRPCR) is utilized with the pink primer (SequencingF) for sequencing.

**Fig 5:**
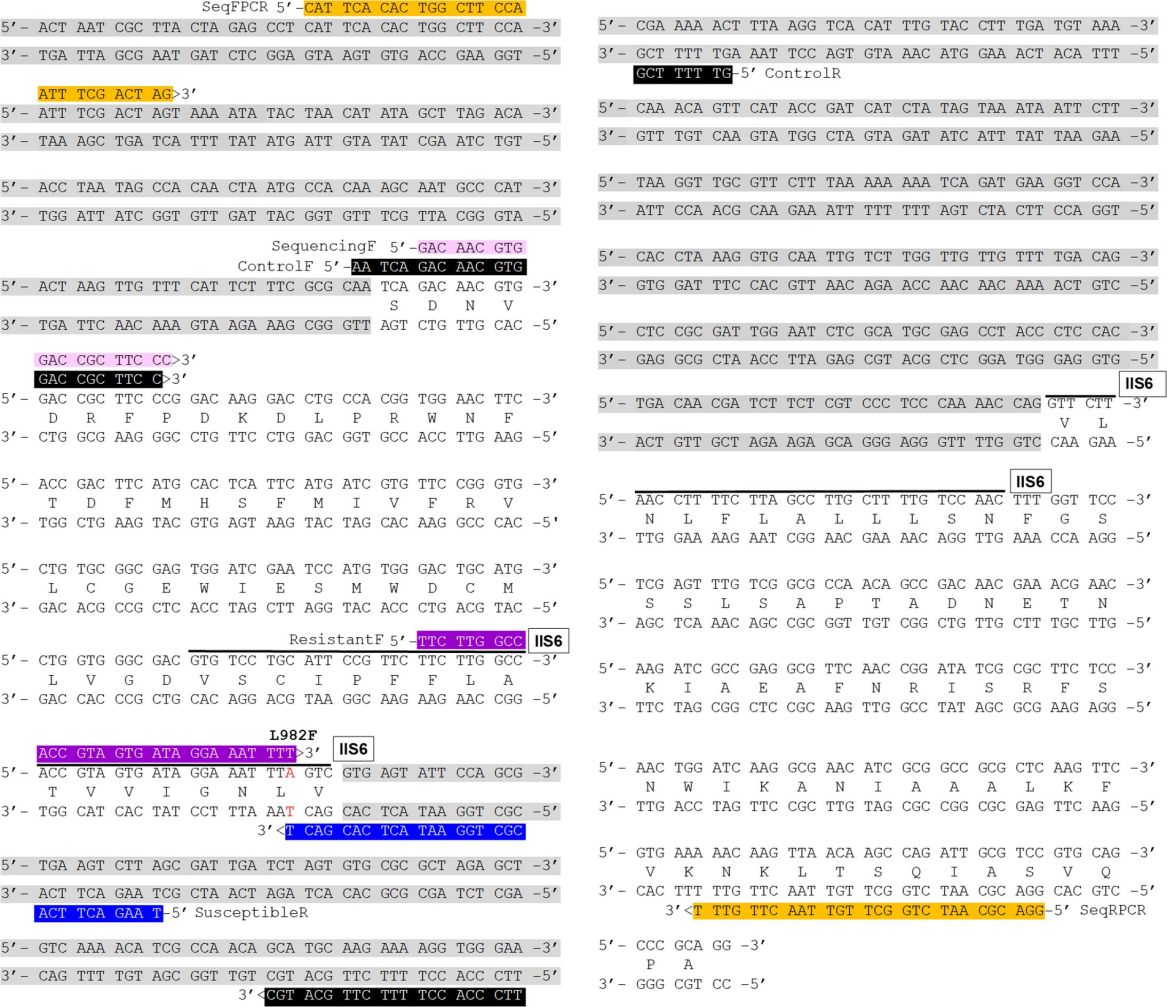
Partial Na_v_ channel sequence with primers for *kdr*-like diagnostic PCR, partial Na_v_ amplification, and sequencing. Intronic regions are in gray. The primer set of diagnostic PCR: control primers (ControlF and ControlR) are boxed in black; the diagnostic resistant primer (ResistantF) is in purple; the diagnostic susceptible primer (SusceptibleR) is in blue. Primers (SeqFPCR and SeqRPCR) for generating an 850 bp PCR product for sequencing are in orange; the primer utilized for sequencing (SequencingF) is in pink.

**Fig 6:**
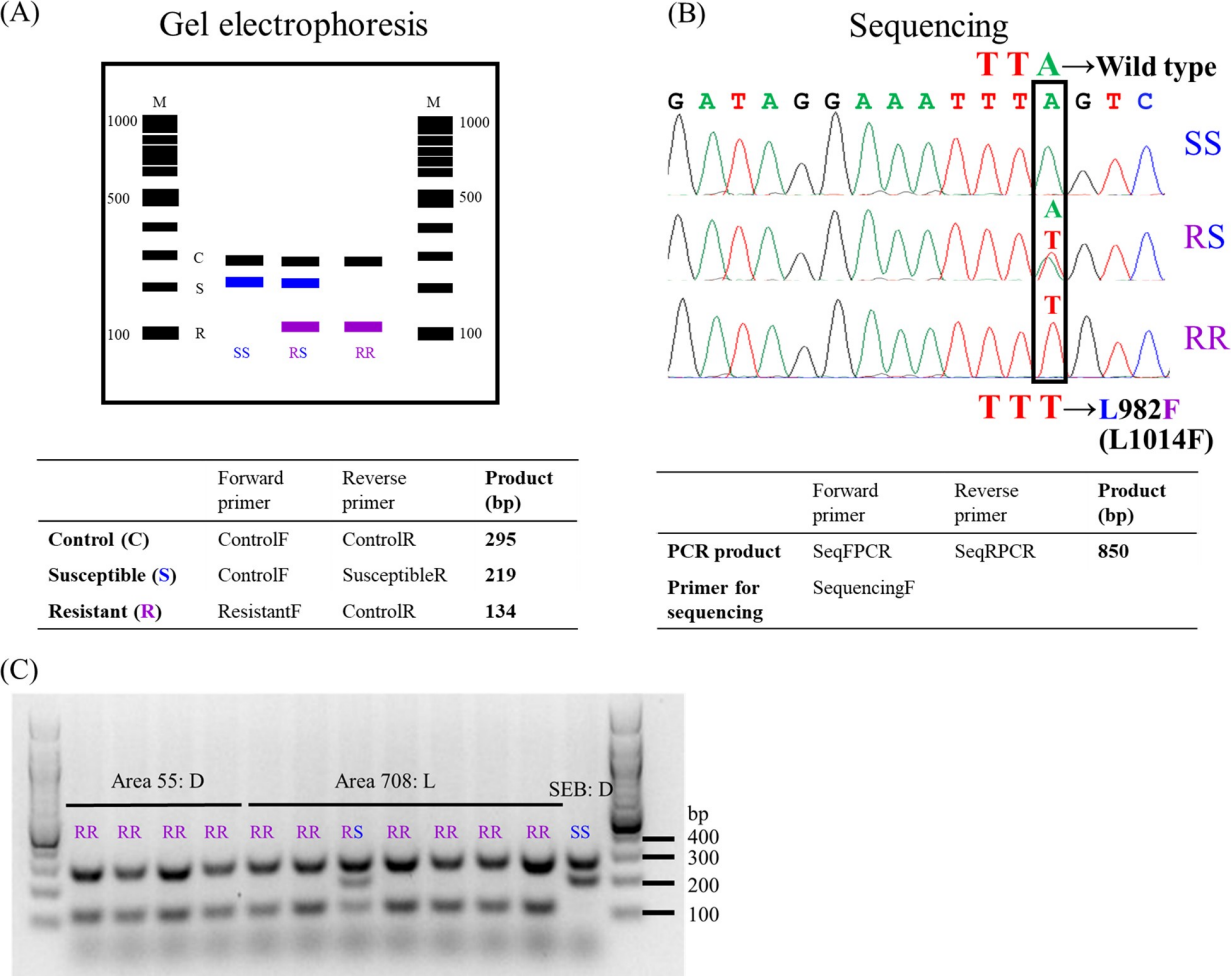
Diagnostic methods for detection of the *kdr*-like mutation in the voltage-gated sodium channel (Na_v_). (A) Schematic of the diagnostic PCR expected products for detecting the single nucleotide polymorphism (SNP) A (susceptible allele) to T (resistant allele), as resolved on a 1.5% agarose gel. The A to T mutation results in the nonsynonymous L982F mutation in the protein. The ControlF and ControlR primers amplify a 295 bp “control band” product (black) that is expected from all mosquitoes (S1 Fig), the ControlF and SusceptibleR primers amplify a 219 bp “susceptible band” product (blue) only if at least one susceptible allele is present, and the ResistantF and ControlR set amplify a 134 bp “resistant band” product (purple) only if at least one *kdr* resistant allele is present. (B) The wild type, susceptible codon TTA changes to TTT in the *kdr* resistant allele (black rectangle) resulting in the sodium channel L982F mutation. Electropherograms of three different genotypes. Top: homozygous susceptible SS (TTA); middle: heterozygous RS (TTA/TTT); and bottom: homozygous resistant mosquitoes RR (TTT) for the L982F mutation. The *kdr*-like mutation amino acid residue position in the sodium channel of the house fly is shown in parenthesis. (C) Diagnostic PCR results of field collected mosquitoes from areas 55 and 708, and Sebring strain (SEB) mosquitoes from 91.2 m. D, mosquitoes were dead after Permanone^®^ treatment; L, alive after Permanone^®^ treatment.

A *kdr*-like allele-specific diagnostic PCR was developed following a comparable strategy for *Anopheles gambiae* reported by Martinez-Torres et al. (1998) [16]. The Na_v_ amino acid sequence of the *Cx*. *quinquefasciatus* S-Lab strain (GenBank: AFQ00696.1) was published by He et al. (2012) [17]. BLAST analysis of this Na_v_ amino acid sequence against the *Cx*. *quinquefasciatus* dataset on VectorBase identified that the Na_v_ gene is encoded in the reverse strand of the gene CPIJ017894, present in the genomic supercontig 3.1170. Alignment of this nucleotide sequence with Na_v_ sequences of *Musca domestica*, *Aedes aegypti*, *Aedes albopictus*, and *A*. *gambiae* helped identify the *kdr*-like mutation site in the *Cx*. *quinquefascitus* gene. Based on the CPIJ017894 gene sequence, primers SeqFPCR and SeqRPCR (Table 1) were designed on intron 18 and exon 20, respectively, for generating a 850 bp PCR product, which would include the *kdr*-like mutation in exon 19 (Figs 4 and 5 and S1 Fig). This product was sequenced. Ten such PCR product sequences obtained from Harris Co. field-collected mosquitoes and 1 Sebring strain mosquito were aligned by CLUSTALW with MEGA X [18]. A 153 bp sequence at the beginning of the intron 19 was found identical among the ten sequences and therefore, used to design a ControlR primer, while the ControlF primer was designed encompassing the end of intron 18 and the beginning of exon 19 (Figs 4 and 5). The susceptibleR primer was designed nested to the ControlR primer. The 295 bp control band is generated by ControlF and ControlR primers, and encompasses the exon 19 containing the *kdr*-like mutation site (Figs 4 and 5 and S1 Fig). The *kdr*-like mutation diagnostics (Fig 6A) is based on the differential perfect binding of the last 3’ nucleotide of the susceptibleR (T) or resistantF (T) primers to the site of the corresponding single nucleotide mutation, TTA in the susceptible allele, or TTT in the resistant allele (Fig 6B). The interaction with the DNA polymerase at the primer:template junction during DNA polymerization initiation is important for the overall amplification [19]. For genotyping, the susceptible allele will be determined by the presence of a 219 bp band, and the resistant allele by the presence of a 134 bp band (Fig 6A and 6C). Detection of the control band will be a pre-requisite to score a mosquito, as it would indicate the acceptable quality of the gDNA, specifically in that region of the Na_v_ gene.

**Table 1:** Primers for *kdr*-like diagnostic PCR and Na_v_ gene fragments amplification and sequencing.

Name	Sequence (5’→3’)	Genome nucleotide position^Φ^ (VectorBase: CPIJ017894)	Purpose	Final conc. [nM]
ControlF	AAT CAG ACA ACG TGG ACC GCT TCC	A^92919^-C^92896^	Diagnostic PCR	125
ControlR	GTT TTT CGT TCC CAC CTT TTC TTG CAT GC	C^92628^-G^92653^	Diagnostic PCR	150
ResistantF	TTC TTG GCC ACC GTA GTG ATA GGA AAT TTT	T^92758^-A^92729^	Diagnostic PCR	150
SusceptibleR	TAA GAC TTC ACG CTG GAA TAC TCA CGA CT	A^92701^-A^92729^	Diagnostic PCR	250
SeqFPCR	CAT TCA CAC TGG CTT CCA ATT TCG ACT AG	C^93037^-G^93009^	Amplify PCR products to be sequenced	500
SeqRPCR	GGA CGC AAT CTG GCT TGT TAA CTT GTT T	G^92188^-T^92215^	Amplify PCR products to be sequenced	500
SequencingF	GAC AAC GTG GAC CGC TTC CCA A	G^92914^-C^92895^	Sequencing	5000

^Φ^ The reverse strand of the annotated gene, CPIJ017894, in the genome SuperContig 3.1170 in VectorBase encodes a partial sequence of the *Cx*. *quinquefasciatus* Na_v_ channel (sense strand) (see S1 Fig). The nucleotide positions shown here are as numbered in VectorBase.

### *Kdr-*like mutation diagnostics by Allele-Specific PCR (AS-PCR)

Reactions for diagnostic PCR of individual females to identify the *kdr-*like L982F mutation in the Na_v_ gene were performed with 40 ng of gDNA and four different primers, ControlF, ResistantF, SusceptibleR, and ControlR. Primer sequences and final concentrations are shown in Table 1. GoTaq^®^ master mixes (Promega, Madison, WI) (12.5 μl) and water were added, for a final volume of 25 μl. PCR cycles were: 94°C for 3 min of initial denaturation, 25 cycles of: 94°C 30 s, 62°C 30 s, 72°C 60 s, and a final 72°C 5 min extension step. Products were resolved in a 1.5% agarose (Agarose I™, standard melting; VWR Life Science, Missouri, TX) gel with 1X TAE buffer and 10,000 x GelStar™ nucleic acid gel stain (Lonza, Lake Charles, LA), ran at 75 V for 55 min.

### *Kdr*-like mutation diagnostics by sequencing

To determine the error rate of the diagnostic PCR, a subset of the females (n = 79) that had been analyzed by diagnostic PCR were additionally used to generate the above-mentioned PCR product of 850 bp amplified from gDNA to be sequenced (Figs 4, 5 and 6B). Each amplification reaction consisted of 40 ng of gDNA, primers SeqFPCR and SeqRPCR each at 500 nM, 200 μM dNTPs, Phusion Green High-Fidelity DNA polymerase (1 μl = 2U; Thermo Scientific, Waltham, MA), 10 μl Phusion green buffer, and nuclease free water to 50 μl. PCR cycles were as follows: 98°C for 30 s initial denaturation, 35 cycles of 98°C 10 s, 63°C 10 s, 72°C 45 s, and 10 min final extension at 72°C. Additionally, a single nested forward primer for sequencing, SequencingF, was designed to the 3’ of primer SeqFPCR (Table 1, Figs 4 and 5). PCR products were separated by 1.5% agarose gel electrophoresis (Agarose I™) with 1X TAE buffer, run at 75 V for 55 min, and visualized with GelStar™ nucleic acid gel stain (5 μl stain/50 ml agarose gel; Lonza). The ladder was ZR 100 bp DNA Marker (Ready-to-Load; 2 μl/ well; ZYMO Research). PCR products were excised from the gel with a razor, transferred into 1.5 ml microcentrifuge tubes, and purified with the Zymoclean^TM^ gel DNA recovery kit (ZYMO Research) following the kit instructions. The DNA was eluted in 30 μl of nuclease free water after 5 min of incubation before centrifugation. The concentration of recovered PCR products was measured with a Nanoquant M200 Pro. Each PCR product (200 ng) and 5 μM of sequencing primer per reaction were sent to Eton Bioscience Inc. for sequencing, and sequences were analyzed using Chromas (Technelysium, South Brisbane).

### Statistical analyses

Statistical analyses were performed using SAS^®^ 9.4 (Cary, NC) and R 3.6.3 [20].

The Pearson Chi-square statistic was used for statistical analyses of percent survival of females from different operational areas and distances in the field cage tests. In those cases where the P-value of the Chi-square test was less than 0.05, the data were further analyzed using a Bonferroni corrected Fisher’s Exact test [21,22] for pairwise comparisons to discover any significantly different pairs of operational areas or distances in the field cage tests. These two statistical analyses methods were also applied in the analyses of the *kdr*-like allele frequency of mosquitoes in each area; percent of the two genotypes, *kdr*-homozygous and heterozygous resistant, across the 6 areas; and the percent of two genotypes in the proportion of surviving mosquitoes at different distances. The survival probabilities of the two resistant genotypes were also assessed. The differences among death odds-ratios across 6 areas and 3 distances were analyzed by the Breslow-Day test statistic. In this study, no differences in the death odds-ratios were identified. All odds-ratios were combined to a single value and tested using the Cochran-Mantel-Haenszel (CMH) test statistic for comparing the odds of death for homozygous and heterozygous resistant genotypes [23,24]. The impact of the factors, area, genotype, distance and cage on the probability of survival of the mosquitoes was assessed through a logistic regression model. The model of the log-odds, where p equals probability of survival, is:

log(p/(1−p))=bo+b1*area+b2*genotype+b3*distance+b4*cage+e

The Wald’s test was performed to obtain the confidence intervals for the odds ratios.

The number of field-collected mosquitoes genotyped as homozygous susceptible was considered too low to be included in the analyses of: effect of genotype on survivorship, the proportion of RR and RS among operational areas, the percent survivorship of RR and RS genotypes within each distance, the odds-ratios of survivorship for genotype RR and RS and area and distance, and the logistic regression analysis.

## Results

### Effect of treatment distance on survivorship

**Fig 7:**
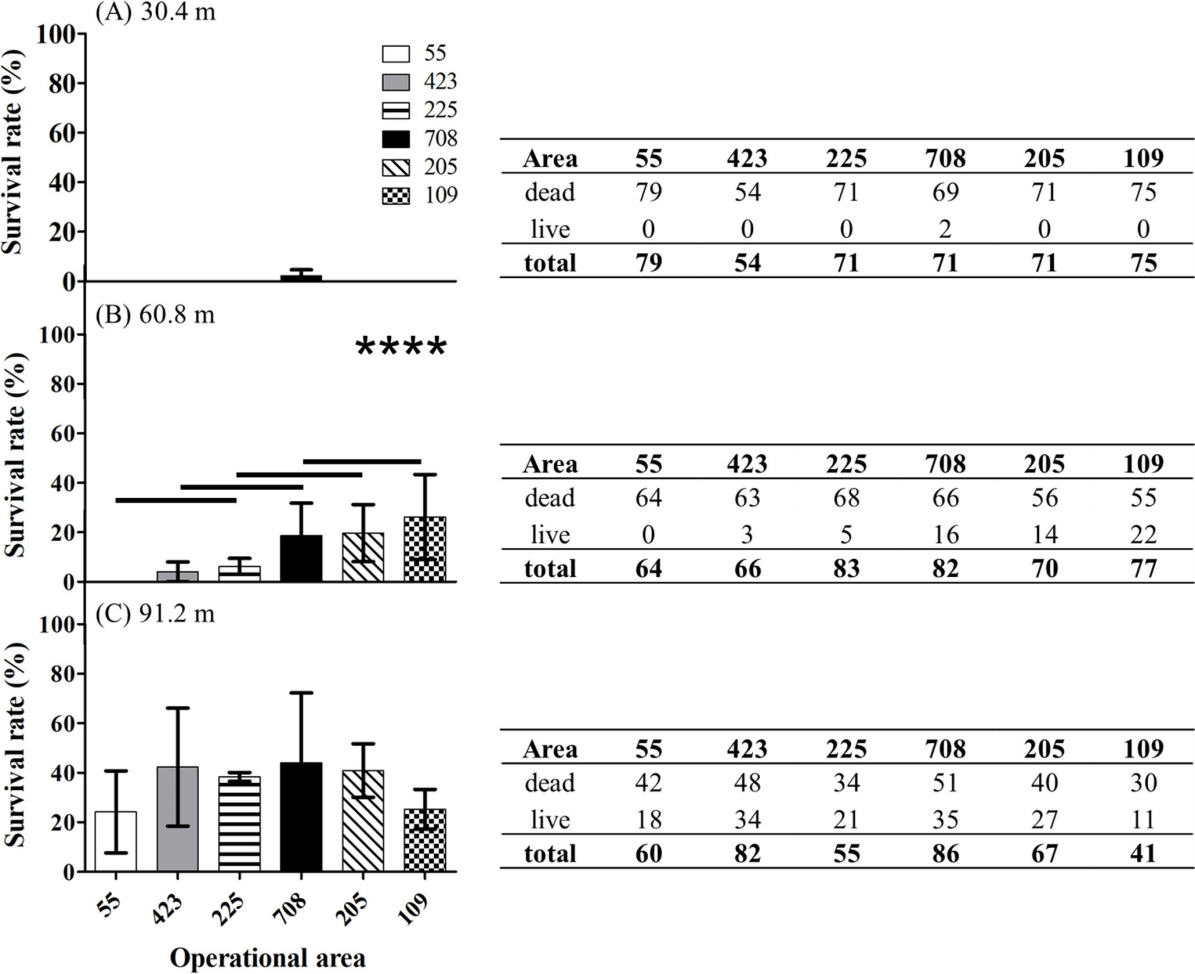
Survival rate of *Cx*. *quinquefasciatus* in the Permanone^®^ field cage tests from six areas. (A) Permanone^®^ 31–66 was applied at 30.4 m from cages; (B) at 60.8 m; or (C) at 91.2 m. The tables on the right present for each distance the number of dead and alive mosquitoes from each area, after field cage tests; these numbers were used for the histograms on the left. The Chi-square test was only significant for the survival rates at 60.8 m with *P* < 0.0001. Therefore, the analyses were followed by pairwise comparison tests to detect significant differences. Horizontal lines above bars indicate the areas which are not significantly different (*P* < 0.05). For each area, histograms show Mean ± SEM.

In total, 1,254 female mosquitoes from 6 operational areas in Harris County were tested in field cage tests. We first asked for each treatment distance if the survivorship of females was different among the six operational areas. At 30.4 m, the majority of the females were dead after treatment with Permanone^®^ 31–66 (non-significant differences; Chi-square, *P* = 0.1056) (Fig 7A). At 60.8 m, the percent survival of females was significantly different among the 6 operational areas (Chi-square, *P* < 0.0001). The pairwise comparisons indicated that the survival of females from area 109 was significantly higher than those from areas 55, 423 and 225; the survival from area 205 was significantly higher than those from area 55 and 423; the survival of females from area 708 was significantly higher than females from area 55 (Fig 7B). At 91.2 m, the survival rates from the 6 different operational areas was not statistically different (Chi-square, *P* = 0.0940) (Fig 7C).

### Genotyping and accuracy rate of the Allele Specific-PCR (AS-PCR) for detecting the *kdr*-like mutation

In total, 1,028 mosquitoes from 6 operational areas in Harris County were genotyped (Table 2); 161 mosquitoes were genotyped by sequencing and 867 mosquitoes were genotyped by AS-PCR. These analyses resulted in 839 RR, 181 RS, and 8 SS (1 SS mosquito was identified by sequencing and the rest by AS-PCR).

**Table 2:** Number of genotyped mosquitoes from each area.

	Area						
	423	708	225	109	55	205	Total
Sequenced	41	53	5	3	56	3	161
Diagnostic PCR	146	180	132	109	137	163	867
**Total**	**187**	**233**	**137**	**112**	**193**	**166**	**1,028**

Based on the comparison of the results for each female of both PCR product sequence and diagnostic PCR we concluded that the diagnostic PCR (primer set and PCR condition) has an overall 97.47% accuracy rate for the detection of *kdr*-like mutation in *Cx*. *quinquefasciatus*. The result of two females diagnosed as homozygous resistant through diagnostic PCR was corrected to heterozygous resistant based on sequencing. The remaining 77 samples, including homozygous and heterozygous resistant and susceptible field females, had congruent results of sequencing and diagnostic PCR. The SEB strain females always yielded the expected sizes of PCR products that were verified by sequencing.

### Spatial distribution of resistant genotypes

**Fig 8:**
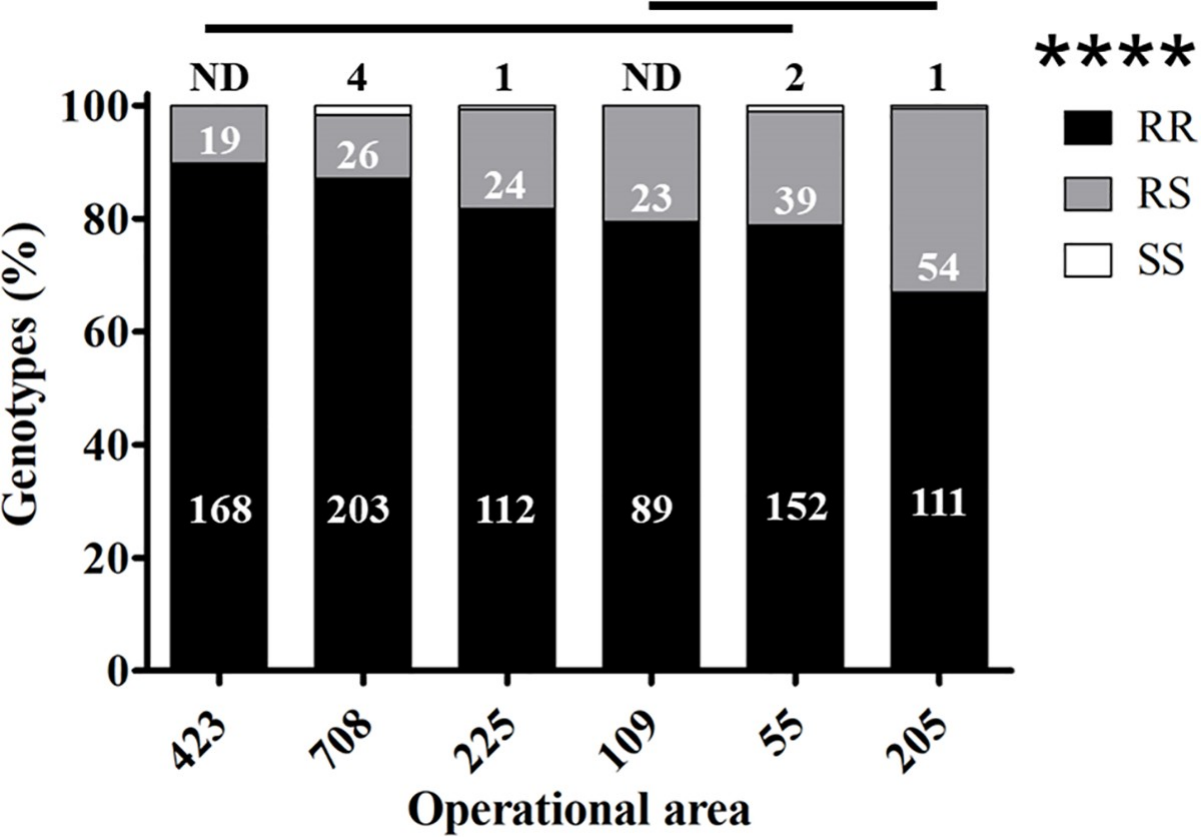
Percent of each genotype (RR, RS, and SS) of *Cx*. *quinquefasciatus* from per area. **** *P* < 0.0001 calculated by Chi-square. Horizontal lines above bars indicate the areas which are not significantly different (*P* < 0.05). The numbers in the graph refer to the numbers of mosquitoes analyzed for each area: in black on the top of each bar are the number of susceptible (SS) mosquitoes; ND = non-detected susceptible mosquitoes; in white in the gray boxes are the number of heterozygous resistant (RS), and in white in black boxes are the number of homozygous resistant (RR).

**Fig 9:**
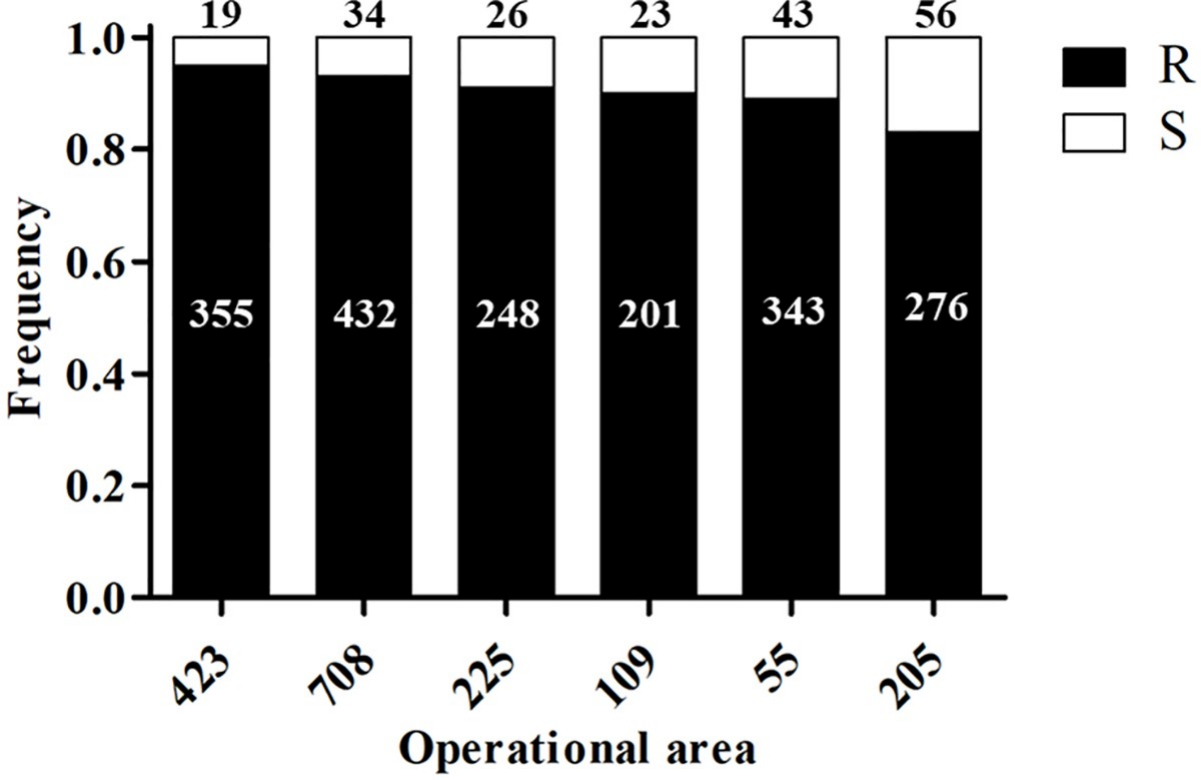
Frequencies of *kdr*-resistant and susceptible alleles of *Cx*. *quinquefasciatus* per area. There were no significant differences among areas (*P* > 0.05). The numbers on bars refer to the numbers of mosquitoes’ alleles analyzed for each area: in black on top are the number of susceptible allele (S), and in white in black boxes are the number of *kdr*-resistant allele (R).

Because we had detected differences in survivorship among areas when the mosquitoes were treated at 60.8 m (Fig 7B), we then asked if the proportion of homozygous for the *kdr*-like mutation allele frequency (RR) and that of heterozygous RS were significantly different among the six areas. The field collected susceptible mosquitoes were not included in the statistical analysis of proportion of genotypes in the six areas, because of the insufficient sample size, as only 8 such females were found. These mosquitoes were also eliminated from the analyses that follow (see Statistical Analysis). The proportion of homozygous resistant (RR) mosquitoes was significantly different among the 6 areas (Chi-square, *P* < 0.0001) (Fig 8). The subsequent pairwise comparisons with Bonferroni correction indicated a lower proportion of homozygous resistant females in area 205 than in areas 423, 708, and 225 (Fig 8). As expected, complementarily, the proportion of heterozygous resistant (RS) mosquitoes among the areas was also significantly different (Chi-square, *P* < 0.0001). The pairwise comparison also indicated a higher proportion of heterozygous resistant mosquitoes in area 205 than 423, 708, and 225 (Fig 8). It followed that the *kdr*-like mutation allele frequency (R) was significantly higher than the susceptible allele (S) (Chi-quare, *P* < 0.0001). In general, the resistant allele frequency is higher than 0.8 across the 6 operational areas. Although there were no significant differences among areas, areas 423, 708, 225, and 109 had a resistant allele frequency higher than 0.9 (Fig 9).

### Effect of genotype on survivorship of *kdr*-resistant homozygous and heterozygous females at each distance

**Fig 10:**
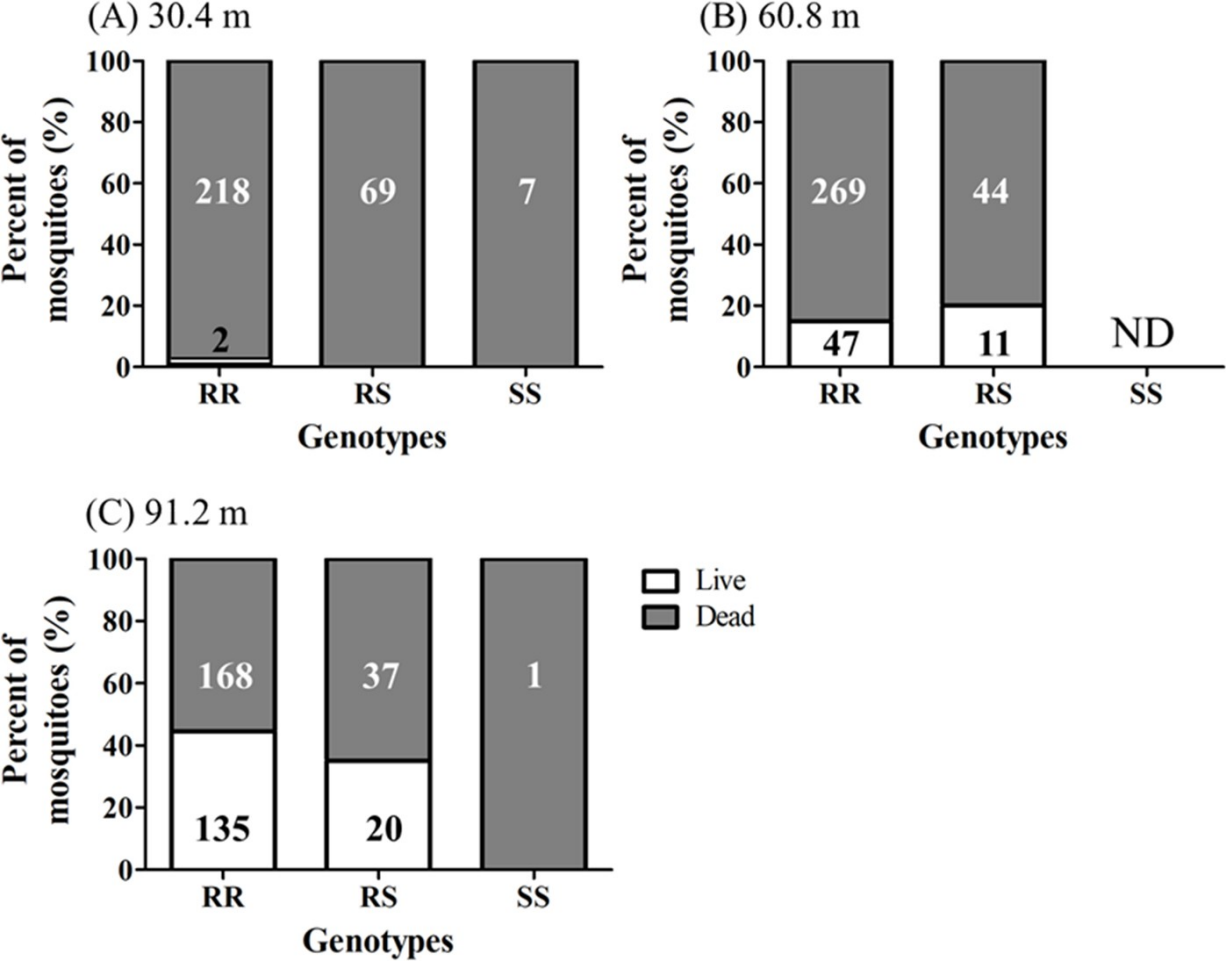
Percent of live or dead mosquitoes from the 6 areas by distances and genotypes. (A) 30.4 m from the point of Permanone^®^ 31–66 application. There were two homozygous resistant females that survived. (B) 60.8 m from the point of Permanone^®^ 31–66 application. (C) 91.2 m from the point of Permanone^®^ 31–66 application. ND = non-detected among field-collected mosquitoes. In white, over the gray zone in each bar, is the number of genotyped dead mosquitoes, and in black over the white zone, the number of genotyped mosquitoes that survived. At each of the three distances, there were no significant differences in the percent of dead mosquitoes depending on their RR or RS genotype (Chi-square tests). While the figure shows the number of susceptible mosquitoes these were not included in the analyses. In addition, results were confirmed by both the Breslow-Day test that found no evidence of a difference in the 11 odds-ratios (*P* = 0.3283) (See text for explanation), and the CMH test allowed to conclude that there were not differences in the odds of death for RR and RS resistant genotypes.

We found that both survivorship of females at 60.8 m of the treatment source, and the proportion of *kdr* homozygous and heterozygous resistant females were significantly different among the areas, we then investigated if the two resistant genotypes, RR and RS, differentially affected females survivorship at each of the three distances, and disregarding areas of origin. As before, the field collected susceptible mosquitoes were not included in the statistical analysis of survivorship of genotypes at three distances. The percentage of surviving mosquitoes was not significantly different between homozygous and heterozygous resistant mosquitoes at 30.4 (Chi-square, *P* = 1.000), 60.8 (Chi-square, *P* = 0.3045) and 91.2 m (Chi-square, *P* = 0.3338) after treatment with Permanone^®^ 31–66 (Fig 10). That is, within each distance, RR and RS mosquitoes survived equally.

### Analysis of factors affecting survivorship in the field

While we found no evidence that genotypes alone, RR or RS, had differential influence in survivorship, but we found that some areas had differential survivorship (e.g. at 60.8 m, areas 708, 205, and 109 with higher survivorship; Fig 7), as well as significantly more homozygous resistant individuals (more homozygous resistant mosquitoes in areas 423, 708, and 225; Fig 8). This led us to inquire if the probability of the survivorship of each genotype could be influenced by the areas of origin and/or the three different treatment distances. For this, the Breslow-Day test of Homogeneity of the Odds Ratios of death was applied across the 6 areas and 3 distances for 18 possible combinations of area and distance. However, the test excluded the mortality results from 30.4 m because all mosquitoes were dead (odds ratios cannot be computed due to a zero value in the denominator), and also it excluded the 100% mortality results of area 55 from 60.8m, reducing the number of combinations with computed odds ratios to 11. This analysis yielded a *p*-value of 0.3283, which indicates no significant evidence of a difference in the odds ratios of death across RR and RS for the 11 combinations of area and distance for which the odds ratios could be estimated. For the 7 area and distance combinations that were excluded from the Breslow-Day test, the probability of survival of RR and RS is zero, the same for both genotypes. Thus, a common odds-ratio of the odds of death across genotypes RR and RS is 0.9654 with 95% C.I. (0.58, 1.60). Thus, there is not significant evidence that the odds of death for RR and the odds of death for RS are different in all 6 areas and 3 distances for which we obtained bioassay results (Fig 10 further illustrate this conclusion).

The failure to reject the null hypothesis of homogeneity of odd ratios allowed us to subsequently conduct the Cochran-Mantel-Haenszel test (CMH) for differences in the proportions of dead across the genotypes RR and RS adjusting for the effects of the 6 areas and 3 distances on mortality. The CMH test resulted in a *p*-value of 0.6803. This indicates that after adjusting for area and distance there is not significant evidence of a difference in the odds of death between the genotypes RR and RS. Therefore, in our field-cage tests both resistant genotypes had equal percentage of survivorship at the high rate of Permanone^®^ 31–66 applied (See also Fig 10).

**Fig 11:**
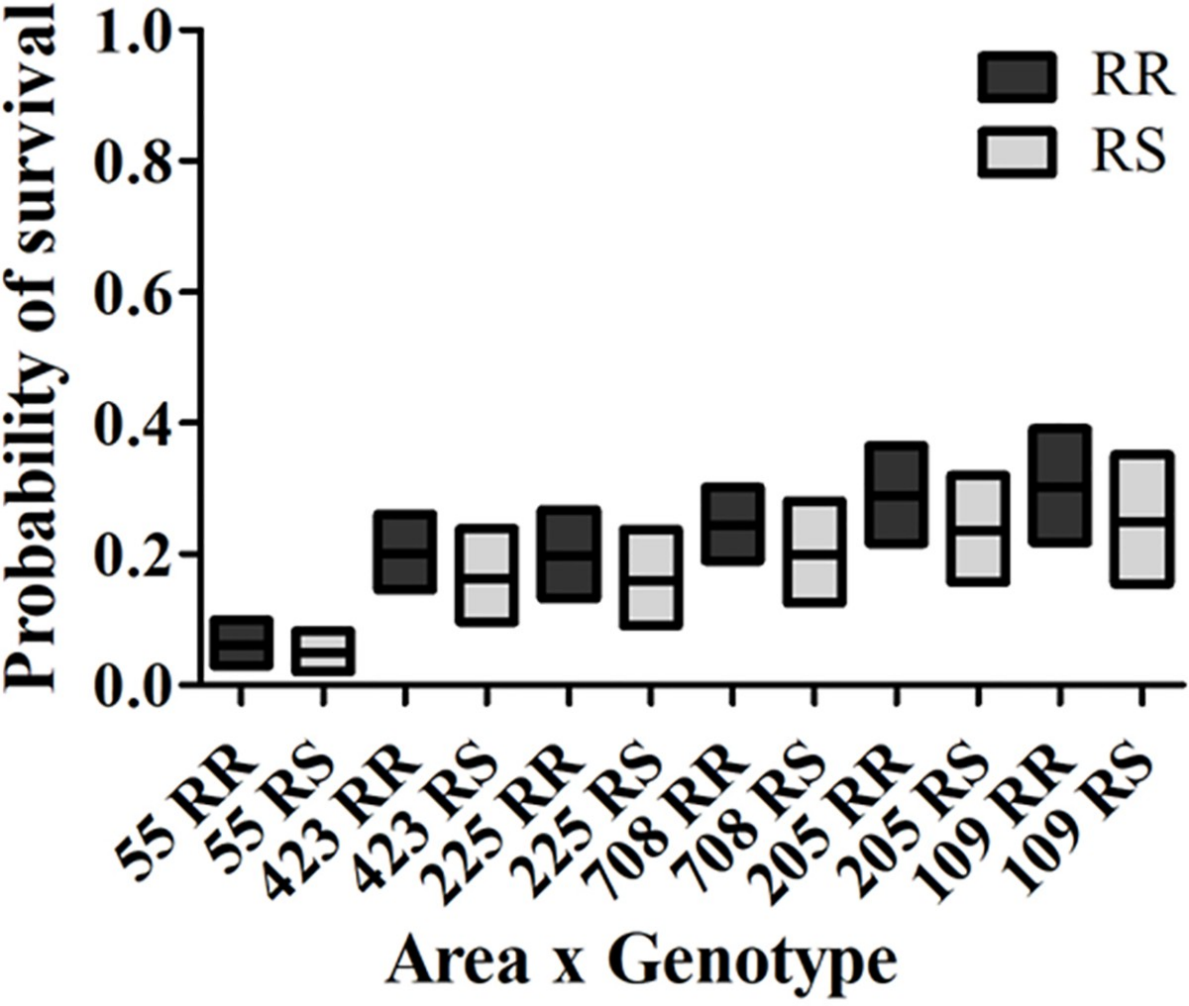
Survival probability of *kdr* homozygous and heterozygous resistant *Cx*. *quinquefasciatus* from the 6 areas. The boxes’ middle lines represent the predicted probability of survival obtained by logistic regression analysis. The top and bottom values of the boxes are the upper and lower confidence intervals (CIs) (S1 Table). The overall model fit (logistic regression, *P* < 0.0001).

The logistic regression model was then fit to the data to obtain estimates of probability of death for mosquitoes for the 6 areas, 3 distances and the two resistant genotypes. The analysis identified area (*P* < 0 .0001), distance (*P* < 0.0001), and cage within distance (*P* < 0 .0001) as significant effects to the survival probability of mosquitoes (logistic regression, *P* < 0.0001). There was not a significant effect of either homozygous or heterozygous resistant genotypes to the estimation of the survival probability of mosquitoes (*P* = 0.5915). The odds ratio of survival for RR vs RS is 1.16 with a 95% Confidence Interval (0.68, 1.942). Because 1 is contained in the interval, the odds of survival for RR is not significantly different from the odds of survival for RS. There is no significant evidence of a difference in the predicted probabilities of survival across the 12 combinations of area and genotype as can be observed in the following plot with 95% confidence intervals (Fig 11).

## Discussion

The *kdr*-like mutation has been associated with resistance to pyrethroid based pesticides in several species [25]. The control efficiency of pyrethroid based pesticides could be estimated through routinely monitor the *kdr*-like mutation in vector population. Diagnostic PCR is a low-cost, simple and highly efficient detection method which could serve this purpose well. Although Martinez-Torres et al. (1999) and Song et al (2007) published AS-PCR for detecting *kdr* gene in *Cx*. *pipiens* complex and *Cx*. *pipiens pallens* respectively, an AS-PCR for *Cx*. *quinquefasciatus* has not yet been developed [13,26]. Chen et al. (2010) also published a set of AS-PCR for *Cx*. *pipiens pallens* [27]. The sequences of these latter primers partially overlap with the those designed in this study. However, the primers published by Chen et al. (2010) are 19–23 bp, while the primers in this study are longer, 24–29 bp, for increasing specificity [27]. Martins et al. (2019) developed an engineered-tail allele-specific-PCR (ETAS-PCR) for detecting L1014F in *Cx*. *quinquefasciatus* [28]. ETAS-PCR diagnostics relies on the design of different tail sequences incorporating an enzyme cutting site, each associated with a specific *kdr*-like mutations. PCR products are identified by their differential sizes after enzymatic digestion. The AS-PCR developed in this study is specific for detecting A^2946^T in *Cx*. *quinquefasciatus* by PCR without other technical requirements.

The *kdr*-like mutation L982F (L1014F) found in the *Cx*. *quinquefasciatus* populations from the 6 operational areas in Harris County is contributed by the nucleotide change from A to T at position 2,946 in the mRNA sequence (position 92,729 in VectorBase: CPIJ017894), with the codon TTA becoming TTT, and consequently causing the amino acid change from leucine to phenylalanine in the voltage-gated sodium channel. The same nucleotide change was recently reported in *Cx*. *quinquefasciatus* collected from operational areas 510 and 515 in Harris County in 2014 [29], and this is the only mutation we detected by sequencing the 850 bp (Fig 6B) band from 161 females from different areas (Table 2). This codon change was also detected in mosquitoes collected from New York, Pennsylvania, North Carolina, South Carolina, Florida, Alabama and California [30–32]. Although one alternative codon change from TTA to TTC for L982F was detected in Ugandan *Cx*. *quinquefasciatus*, so far, L982F caused by the TTA to TTT mutation is the most frequently detected *kdr*-like mutation in the USA [33]. A second *kdr*-like mutation at the same site, L1014S, was detected in the *Cx*. *pipiens* complex (*pipiens* and *quinquefasciatus*) in the USA [31], however, this mutation was not detected in this study. Du et al. (2013) showed that different mutations on the Na_v_ of *Ae*. *aegypti* confer different levels of resistance to pyrethroids type I and type II [34]. The leucine-serine *kdr*-like mutation confers a higher level of resistance to Dichlorodiphenyltrichloroethane (DDT) than to pyrethroids in *Cx*. *pipiens* [13]. The *kdr*-like mutation detected in Harris County of L to F may be preferentially selected by high selection pressure of permethrin-based adulticide because DDT is not applied in the USA. Additionally, the change of L to F or S confers higher resistance to pyrethroids than L to H [35]. Chen et al. (2010) found that L1014S allele frequency decreased in environments with and without deltamethrin and suggested this mutation might not directly confer deltamethrin resistance [27].

Analyses from areas 109, 205 and 225 showed many mosquitoes could not be genotyped in the diagnostic PCR according to our criteria, because the control primers did not yield the expected control band (295 bp), even when in some mosquitoes the putative diagnostic resistant bands amplified. This could be due to having collected eggs from multiple cryptic *Culex* species in these three north areas. The landscape in these areas contain more barren land, grassland and evergreen forest compared to areas 55, 423, and 708 (S2 Fig) [36]. The different habitat composition will cause different abundance of mosquito species [37,38]. *Culex* sp. collected from the six areas (mosquitoes from the upwind control cages) were sent to DSHS for *Culex* species identification. *Culex nigripalpus* was identified from Area 109, potentially explaining the absence of the control band in some females.

The *kdr*-like allele frequency in the *Cx*. *quinquefasciatus* population in Harris County is higher than 0.83 across the 6 areas (Fig 8). Further, mosquito populations in areas 423, 708 and 225 have *kdr*-like allele frequencies higher than 0.9. These three areas received more than 10 pyrethroid based pesticide applications from HCPH-MVCD from 2016 to 2018 (S3 Fig). This high pyrethroid selection pressure could be the reason for the high *kdr*-like allele frequency in these three areas. However, the frequency of the *kdr*-like allele in area 109 of 0.9 is not associated with Permanone^®^ applications conducted by HCPH-MVCD because they did not apply any pyrethroid based adulticide in area 109 from 2015 to 2018. There are at least two possible reasons for the high *kdr*-like allele frequency in this mosquito population without public pyrethroid application records for three years until the time of this study. First, the presence of high *kdr*-frequency in mosquitoes from areas that have not received public pyrethroid applications suggests gene flow across areas. There might also be heavy applications by private contractors with pyrethroid-based adulticides in area 109 and the surrounding areas, such as areas 114 and 112 (Fig 2), or pyrethroids applications to control other pests. The George Bush intercontinental airport is located in these two areas and private contractors handle airport pest control. We were unable, however, to determine the identity of such contractors to identify the active ingredients used. Second, perhaps the homozygous resistant genotype has been established as the major genotype in the mosquito population in area 109 previous to 2015. Theoretically, the frequency of the susceptible allele would recover after a few generations without insecticide selection because the *kdr*-like mutation has shown fitness costs in populations without pesticide selection pressure [10,39,40]. However, if the homozygous resistant genotype is prevalent in the mosquito population in area 109, the recovery of the susceptible allele frequency is likely to be slow [41]. In horn fly populations, alternatively applied insecticides with different mode of actions did not eliminate the established super-*kdr* (*skdr)* and *kdr* pyrethroid resistance genes [42].

In our field cage tests, all females of the *Cx*. *quinquefasciatus* Sebring strain survived when upwind, therefore, the mortality data in the cages downwind did not require correction with the Abbott’s formula [43]. Also, in the treatment zone, all Sebring strain females died at the three distances in both field cage tests, indicating the application was correct and reached the maximal distance (91.2 m; Fig 3). All field-collected females died at 30.4 m except for two from area 708 (Fig 7A). In contrast, around 14% and 35% of the field-collected mosquitoes survived at 60.8 m and 91.2 m, respectively (Fig 7B and 7C). These results suggest the *Cx*. *quinquefasciatus* population in the 6 operational areas in Harris County is resistant to Permanone^®^. These field cage test results can be explained by the high *kdr*-like allele frequency in the field-collected population, as 99% of tested mosquitoes were either homozygous or heterozygous resistant (Fig 8). A previous study with this species showed that after 8 generations of permethrin selection, the percentage of homozygous resistant mosquitoes increased from 38% to 100%. Those permethrin selected mosquitoes were 2,700 fold more resistant than the *Cx*. *quinquefasciatus* S-Lab strain (different from Sebring strain used here) [12]. In our study, the percentage of homozygous *kdr* mosquitoes ranged from 64% for area 205 to 89% for area 423 (Fig 8). This result suggests the mosquito populations in the 6 areas have been under heavy permethrin selection pressure for generations. According to the pyrethroid-based pesticide application record from HCPH-MVCD from 2016 to 2018, only area 109 received no pyrethroid-based pesticide application, the other 5 areas studied herein received 4 to 22 applications of pyrethroid-based pesticide (S3 Fig). Chen et al. (2010) suggested that the *kdr*-like allele frequency is an excellent molecular marker for pyrethroid resistance [27]. Since susceptible mosquitoes of the Sebring strain and those 8 genotyped as susceptible for *kdr*-like mutation died in the field cage test, the *kdr*-like allele is supported as a molecular marker for assessing the pyrethroid-resistant frequency.

Both phenotypic (Fig 7) and genotypic (Figs 8–10) results indicate the existence of a pyrethroid-resistant mosquito population across the 6 operational areas of Harris County. The statistical analyses showed that the probability of survival is not significantly different between homozygous and heterozygous resistant mosquitoes within each distance (Fig 10), the Breslow-Day test did not detect interaction between areas, distances and 2 genotypes, and the linear regression modeling showed that the probability of survivorship of both resistant genotypes is similar across the 6 areas (Fig 11). This partially explains why some mosquito survival rates did not correlate with the proportion of homozygous and heterozygous resistant *kdr* genotypes. For example, the percentage of surviving mosquitoes from area 205, which has the highest number of heterozygous resistant (Fig 8), is significantly higher than the percentage of surviving mosquitoes from area 423 (Fig 7B), which has higher percentage of homozygous resistant. A similar situation has been identified for areas 109, 205 and 708. The survival rates of mosquitoes collected from these three areas are not significantly different (Fig 7B), nevertheless, less homozygous resistant mosquitoes were detected from area 205 than mosquitoes collected from area 109 and 708 (Fig 8). Because the probability of survival of RR is similar to RS, this could be interpreted as one *kdr*-like allele contributing similarly as two *kdr*-like alleles to the survival of *Cx*. *quinquefasciatus* in the field under the currently applied rate of Permanone^®^. However, it has been reported that resistance to pyrethroids is usually incompletely recessive based on probit analysis results [44–46]. The phenotypic resistance may not only be contributed by L982F but also other associated target site mutations, and metabolic resistance [47–49]. Aside from L982F, A109S and W1573R are the two other nonsynonymous *kdr*-like mutations found in the voltage-gated sodium channel in *Cx*. *quinquefaciatus* (Fig 1). Xu et al. (2012) suggested W1573R contributes higher permethrin-resistance than L982F; the presence of other mutations could help clarify the apparent dominance of the *kdr* L982F found here in the Harris County population (Figs 1, 10 and 11) [12]. Mosquitoes from areas 708, 205, and 109 are candidates for further analyses of additional resistance mechanisms, based on the fact that these mosquitoes have higher survival rates at 60.8 m in the field cage tests (compare Fig 7B with Fig 8). For metabolic resistance, cytochrome P450 monooxygenases, e.g. CYP4, CYP6 and CYP325, contribute to permethrin resistance [50]. These target site mutations and metabolic resistance-related genes should be included in future analyses for precisely assessing resistance mechanisms to Permanone^®^ in *Cx*. *quinquefasciatus* from Harris County. It is most likely, however, that the maximal rate of applied Permanone^®^ is sufficient to overcome the fitness advantage of resistant mosquitoes (e.g. either one or two *kdr*-alleles, and/or other resistant mechanisms) if it reaches the females, as distance was a significant effect in explaining survivorship in the field. This study investigated how *kdr*-like mutation affected survival rates of *Cx*. *quinquefasciatus* at three different distances from the Permanone^®^-spraying source. However, the exact resistance level of individual field-collected *Cx*. *quinquefasciatus* from Harris County has not been yet analyzed. To fully assess resistance, planned studies will identify the thresholds of Permanone^®^ that kill susceptible and *kdr*-resistant mosquitoes through Probit analyses.

**Fig 12:**
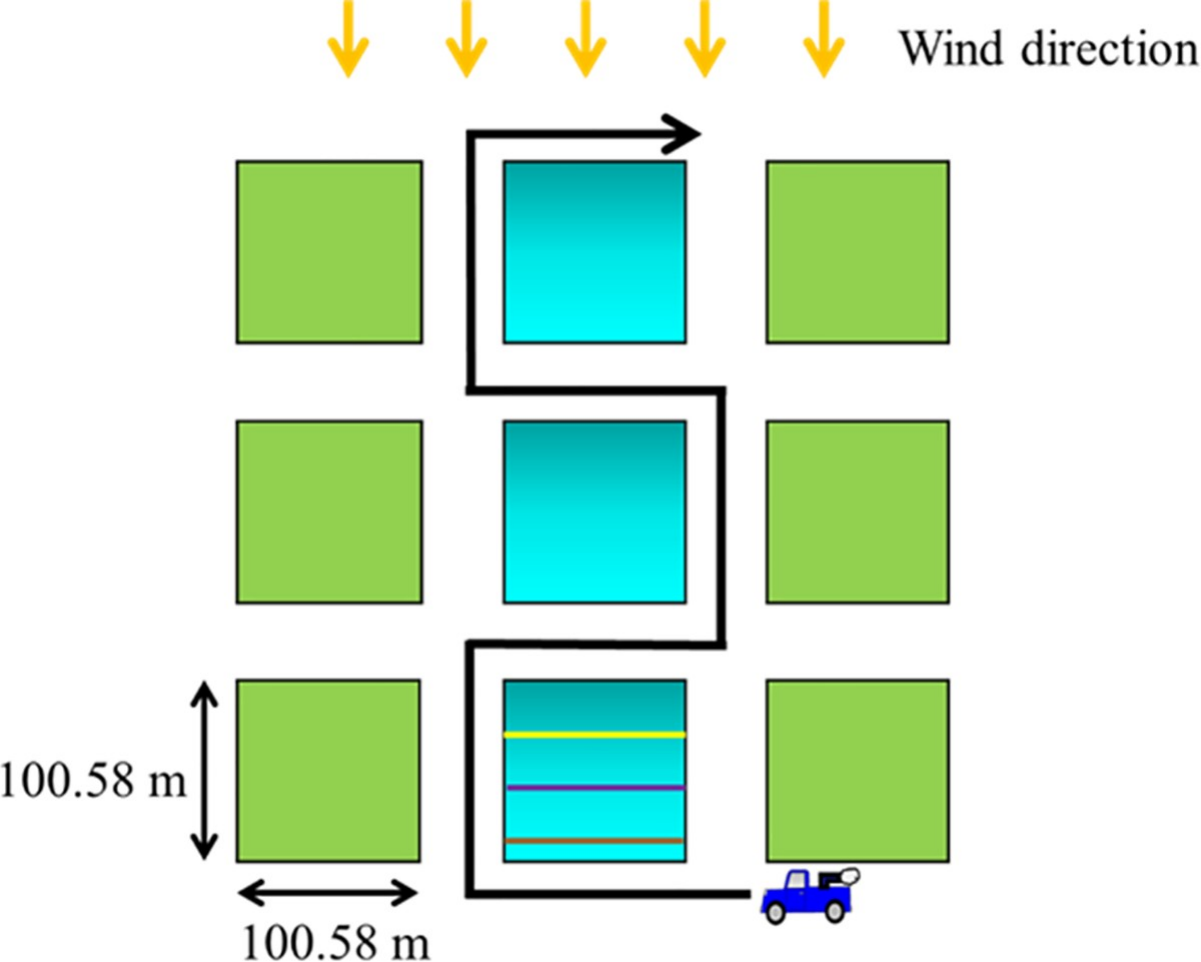
Schematic of pesticide application route in Harris County. The average length of blocks (colored squares) in Houston is 100.58 m. The orange arrows indicate the wind direction. The black single arrow line shows the route of the pesticide application truck. The green squares are the blocks that have not received pesticide application. The squares with blue gradient are the blocks that have received the pesticide application; based on the bioassays results, the darker blue region receives more pesticide droplets than the lighter blue region. The three different colored lines in the gradient blue block indicates the three different distances, 30.4 (yellow line), 60.8 (purple line), and 91.4 (brown line) meters, from the pesticide application source.

The logistic regression model failed to find differences in the survival probability of RR or RS genotypes in the six analyzed areas, indicating that from the Permanone^®^ control perspective the *kdr*-carrying mosquitoes behave as a single population (Fig 11). Permanone^®^ at the currently applied maximum rate can fully control mosquitoes at close distance, 86% at medium distance, and 65% at long distance. This 91.2 m maximal distance tested, roughly corresponds to the average length of a block in Houston (100.58 m). The current design of the application route perpendicular to the predominant wind direction (Fig 12), allows the insecticide to travel down wind, avoids the driving through the pesticide application and yields the most complete coverage. However, the maximum effective range is likely shorter in areas with obstructions, due to settling/ particle sedimentation, impinging on obstacles and droplet dispersal/cloud break up. Therefore, it is likely that the zone farthest from the application route would allow the survival of 14–35% *kdr*-like mutated females (Figs 7 and 12). Despite of this, the treatment route is so designed to prevent areas receiving overlapping applications which would exceed the maximal allowable rate. The future inclusion of two additional distances in the field-cage test at 45.6 m and 76.0 m from the pesticide source would allow a better fit of the logistic regression curve relating probability of death to distance.

Based on our findings, the public health of Harris County will have a higher risk of arboviral disease prevalence if these *kdr*-resistant mosquitoes had higher vector competence when compared to susceptible mosquitoes. For example, *Cx*. *quinquefasciatus* resistant to OPs insecticides can better transmit WNV [51]. The sensitivity of resistant mosquitoes to repellents needs attention as well. *Anopheles* mosquitoes resistant to pyrethroids and organophosphates have different responses to N,N-diethyl-3-methyl benzamide (DEET) from susceptible mosquitoes [52]. To better assess the risk of WNV transmission by *Cx*. *quinquefasciatus*, research on influence of resistance on vector competence and on the efficiency of repellents are needed.

## Supporting information

S1 Fig A schematic of the *Cx*. *quinquefasciatus* Na_v_ gene and detail of the region containing the *kdr*-like mutation (VectorBase supercontig CPIJ017894), and location of primers for diagnostics and sequencing. The schematic of the *Cx*. *quinquefasciatus* VGSC (CxNa-Lv1) gene structure as encoded in the reverse strand, showing 33 predicted exons (light blue boxes) (He et al., 2012). The VectorBase contig CPIJ017894 (46.31 kb) shown below, encodes 13 exons (blue boxes), and 12 introns marked as red lines. This contig encodes the *kdr*-like mutation in exon 2 corresponding to exon 19 in the CxNa-Lv1. The expanded black box below includes the details of the allele specific PCR. The numbers near each primer indicate their sequence location on CPIJ017894, from intron 1 to exon 3. The *kdr*-like mutation TTT (L982F) is marked as a vertical purple band in exon 2. Primers for generating the 850 bp PCR product are in orange; the primer used for sequencing is in pink. The primers for generating the control band for the diagnostic PCR are in black. The blue primer is for detecting the susceptible allele, and the purple primer is for detecting the resistant allele. Expected sizes of corresponding diagnostic bands amplified with these primers are also shown.
(TIF)

S2 Fig Land cover of the six operational areas in Harris County, TX in 2016. (A) Area 109. (B) Area 205. (C) Area 225. (D) Area 708. (E) Area 55. (F) Area 423. The legend below the six figures indicates the land cover category corresponding to each color. The land cover resolution is 30 m. The scale of the figures is not comparable. These images were generated with the publicly available 2016 National Land Cover Database (NLCD) (https://www.mrlc.gov/).
(TIF)

S3 Fig Number of treatments with permethrin-based adulticides, Evoluer^®^ and Permanone^®^ 31–66, in operational areas of Harris County from 2016 to 2018. Mosquitoes were collected from areas delineated by a bold boundary for field cage tests performed in 2018. The layers for county and operational areas boundaries are as described under Fig 2. The map was created with ArcGIS (https://www.arcgis.com/index.html).
(TIF)

S1 Table Predicted survival probability of homozygous and heterozygous resistant *Cx*. *quinquefasciatus* collected from the 6 operational area generated by logistic regression analysis and used to construct Fig 11. P-PrS, predicted probability of survival. CI, confidential interval.
(PDF)
