# Supplementary figures and images for: Detection of the Nav channel kdr-like mutation and modeling of factors affecting survivorship of Culex quinquefasciatus mosquitoes from six areas of Harris County (Houston), Texas, after permethrin field-cage tests
Source: PLoS Negl Trop Dis. 2020 Nov 19;14(11):e0008860. doi: 10.1371/journal.pntd.0008860 (PMC7714350; doi:10.1371/journal.pntd.0008860)

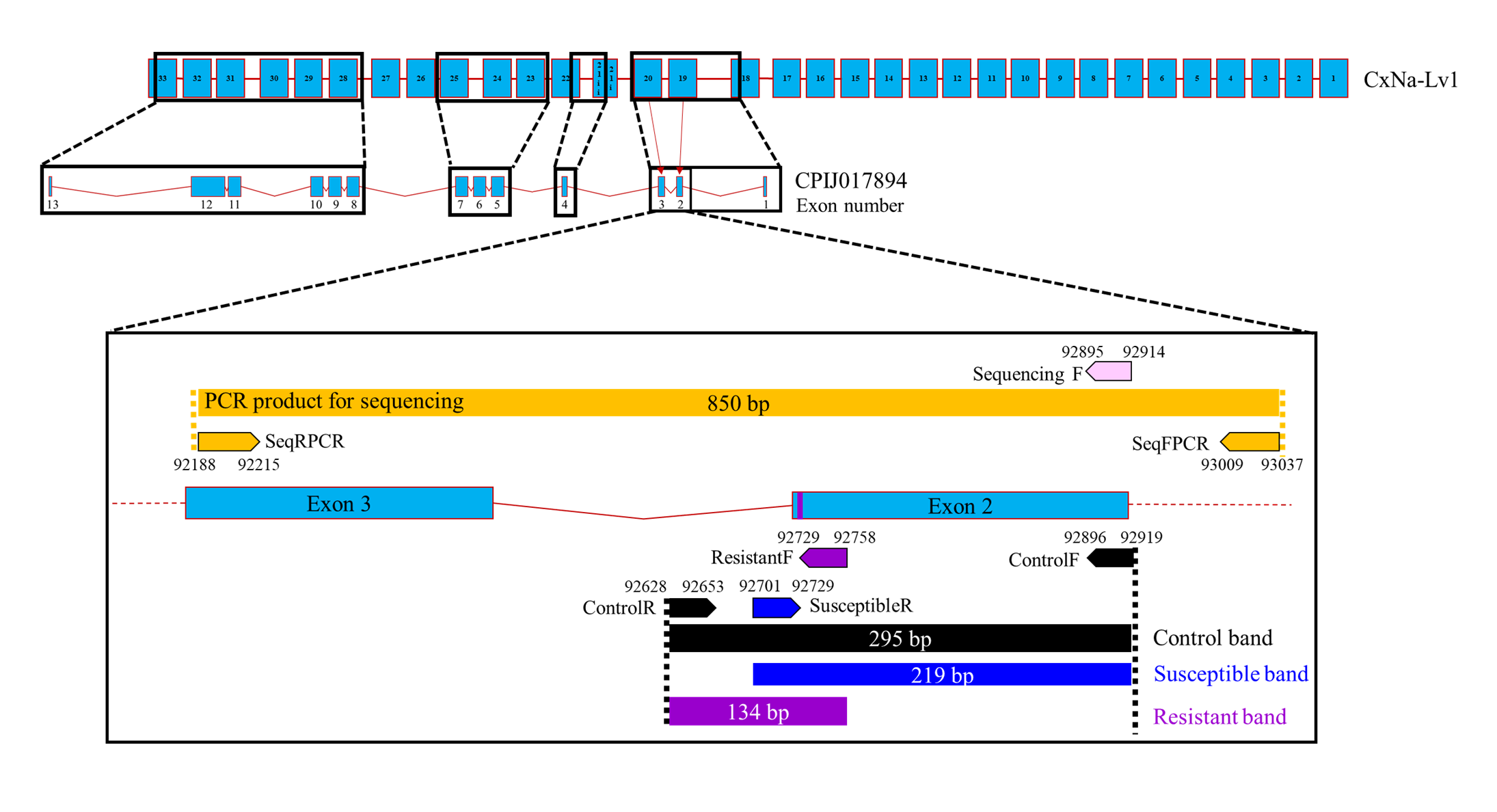

Supplement: S1 Fig — The schematic of the Cx. quinquefasciatus VGSC (CxNa-Lv1) gene structure as encoded in the reverse strand, showing 33 predicted exons (light blue boxes) (He et al., 2012). The VectorBase contig CPIJ017894 (46.31 kb) shown below, encodes 13 exons (blue boxes), and 12 introns marked as red lines. This contig encodes the kdr-like mutation in exon 2 corresponding to exon 19 in the CxNa-Lv1. The expanded black box below includes the details of the allele specific PCR. The numbers near each primer indicate their sequence location on CPIJ017894, from intron 1 to exon 3. The kdr-like mutation TTT (L982F) is marked as a vertical purple band in exon 2. Primers for generating the 850 bp PCR product are in orange; the primer used for sequencing is in pink. The primers for generating the control band for the diagnostic PCR are in black. The blue primer is for detecting the susceptible allele, and the purple primer is for detecting the resistant allele. Expected sizes of corresponding diagnostic bands amplified with these primers are also shown. (TIF) [file pntd.0008860.s001.tif]

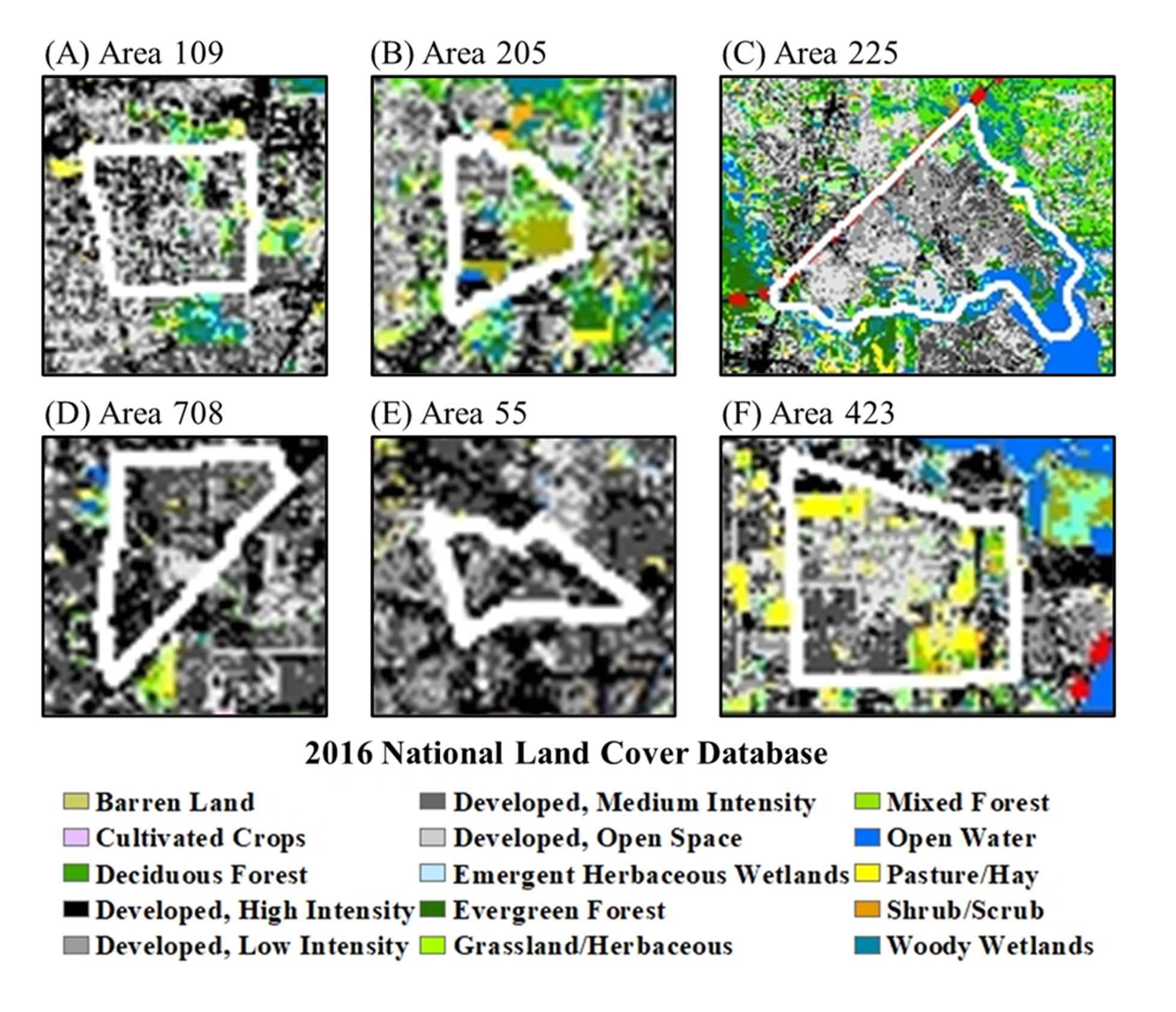

Supplement: S2 Fig — (A) Area 109. (B) Area 205. (C) Area 225. (D) Area 708. (E) Area 55. (F) Area 423. The legend below the six figures indicates the land cover category corresponding to each color. The land cover resolution is 30 m. The scale of the figures is not comparable. These images were generated with the publicly available 2016 National Land Cover Database (NLCD) (https://www.mrlc.gov/). (TIF) [file pntd.0008860.s002.tif]

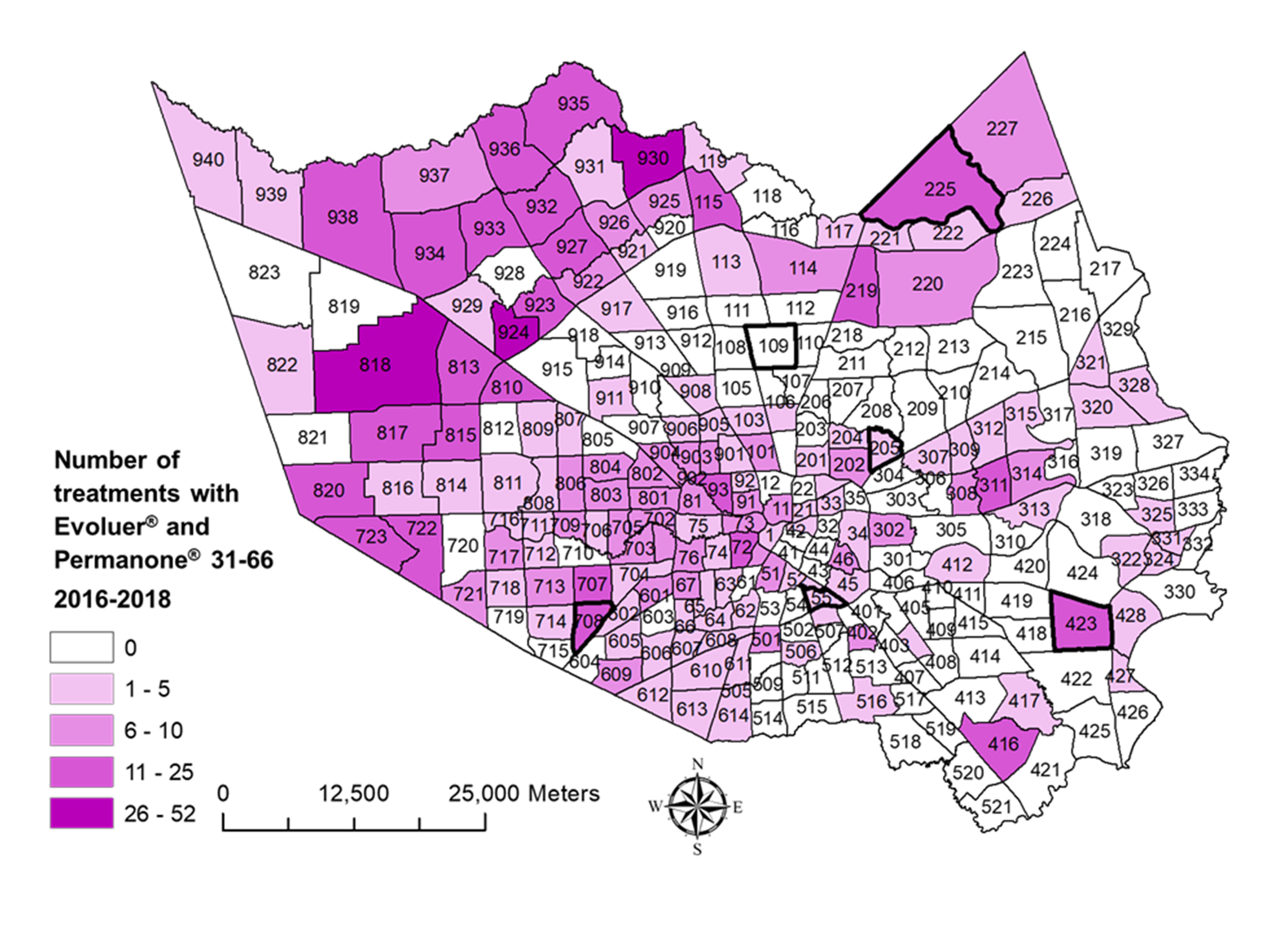

Supplement: S3 Fig — Mosquitoes were collected from areas delineated by a bold boundary for field cage tests performed in 2018. The layers for county and operational areas boundaries are as described under Fig 2. The map was created with ArcGIS (https://www.arcgis.com/index.html). (TIF) [file pntd.0008860.s003.tif]
