# Supplementary material for: Detection of the Nav channel kdr-like mutation and modeling of factors affecting survivorship of Culex quinquefasciatus mosquitoes from six areas of Harris County (Houston), Texas, after permethrin field-cage tests
Source: PLoS Negl Trop Dis. 2020 Nov 19;14(11):e0008860. doi: 10.1371/journal.pntd.0008860 (PMC7714350; doi:10.1371/journal.pntd.0008860)
Supplement: S1 Table — P-PrS, predicted probability of survival. CI, confidential interval. (PDF) [file pntd.0008860.s004.pdf]

S1 Table. Predicted survival probability of homozygous and heterozygous resistant *Cx. quinquefasciatus* collected from the 6 operational areas generated by logistic regression analysis and used to construct Fig. 11.

	Area											
	55		423		225		708		205		109	
Genotype	RR	RS	RR	RS	RR	RS	RR	RS	RR	RS	RR	RS
P-PrS	0.055	0.042	0.197	0.154	0.191	0.150	0.241	0.192	0.285	0.229	0.298	0.240
Lower CI	0.030	0.020	0.145	0.096	0.132	0.090	0.190	0.126	0.216	0.157	0.218	0.155
Higher CI	0.100	0.083	0.261	0.239	0.268	0.238	0.302	0.281	0.366	0.321	0.392	0.352

P-PrS, predicted probability of survival. CI, confidential interval.
